# Selection and validation of reference genes for qRT‐PCR analysis during fruit ripening of red pitaya (*Hylocereus polyrhizus*)

**DOI:** 10.1002/2211-5463.13053

**Published:** 2021-10-06

**Authors:** Qianming Zheng, Xiaoke Wang, Yong Qi, Yuhua Ma

**Affiliations:** ^1^ Institute of Pomology Science Guizhou Provincial Academy of Agricultural Sciences Guiyang China

**Keywords:** fruit ripening, *Hylocereus polyrhizus*, qRT‐PCR, red pitaya, reference gene, RNA‐seq

## Abstract

Red pitaya (*Hylocereus polyrhizus*) is widely cultivated in southern and southwestern China. To provide a basis for studying the molecular mechanisms of the ripening of this fruit, we carried out RNA sequencing (RNA‐seq) analysis to identify differentially and stably expressed unigenes. The latter may serve as a resource of potential reference genes for normalization of target gene expression determined using quantitative real‐time PCR (qRT‐PCR). We selected 11 candidate reference genes from our RNA‐seq analysis of red pitaya fruit ripening (*ACT7*, *EF‐1α*, *IF‐4α*, *PTBP*, *PP2A*, *EF2*, *Hsp70*, *GAPDH*, *DNAJ*, *TUB* and *CYP*), as well as *β‐ACT*, which has been used as a reference gene for pitayas in previous studies. We then comprehensively evaluated their expression stability during fruit ripening using four statistical methods (GeNorm, NormFinder, BestKeeper and DeltaCt) and merged the four outputs using the online tool RefFinder for the final ranking. We report that *PTBP* and *DNAJ* showed the most stable expression patterns, whereas *CYP* and *ACT7* showed the least stable expression patterns. The relative gene expression of red pitaya sucrose synthase and 4, 5‐dihydroxyphenylalanine‐extradiol‐dioxygenase as determined by quantitative real‐time PCR and normalized to *PTBP* and *DNAJ* was consistent with the RNA‐seq results, suggesting that *PTBP* and *DNAJ* are suitable reference genes for studies of red pitaya fruit ripening.

## Graphical abstract

RNA sequencing analysis was applied to study molecular mechanisms of fruit ripening of red pitaya. The generated stably expressed unigenes are a source of potential reference genes for quantitative real‐time PCR. Herein, the expression stabilities of 12 candidate reference genes were evaluated. Polypyrimidine tract‐binding protein and Chaperone protein dnaJ 49 are suggested as suitable reference genes for quantitative real‐time PCR during red pitaya fruit ripening.

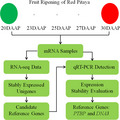

**Abbreviations**
*ACT*
: Actin
Ct: cycle threshold
CV: coefficient of variation
*CYP*
: Cyclophilin
DAAP: days after artificial pollination
ddH_2_O: double distilled water
*DNAJ*
: Chaperone protein dnaJ
*DOD*
: 4, 5‐dihydroxyphenylalanine‐extradiol‐dioxygenase
DOPA: dihydroxyphenylalanine
*EF‐1α*
: elongation factor 1‐α
*EF2*
: elongation factor 2
FPKM: fragments per kilobase of exon per million reads mapped
*GAPDH*
: glyceraldehyde‐3‐phosphate dehydrogenase
*Hsp70*
: Heat shock protein 70
*IF‐4α*
: eukaryotic initiation factor 4‐α
*PP2A*
: serine/threonine protein phosphatase 2A
*PTBP*
: polypyrimidine tract‐binding protein‐like
qRT‐PCR: quantitative real‐time PCR
RNA‐seq: RNA sequencing
SD: standard deviation
*S* *U* *S* *Y*
: sucrose synthase
*TUB*
: Tubulin beta‐8 chain‐like

Quantitative real‐time PCR (qRT‐PCR) is regarded as the most common high‐throughput technique in molecular biology research due to its speed, high sensitivity, reproducibility and specificity [1, 2, 3], and it is widely used to quantify gene expression levels. In qRT‐PCR experiments, reliable internal reference genes are critical for the accuracy and reliability of the quantification of target gene expression levels. The introduction of suitable reference genes can reduce the influence of sample amount, RNA content and integrity, enzyme efficiency of reverse transcription and cDNA quality on the accuracy of experimental results [4, 5, 6]. Generally, the reference genes should have stable expression patterns in different tissues, developmental stages or environmental conditions [7]. Traditionally, housekeeping genes, such as Actin (*ACT*), 18S ribosomal RNA, glyceral‐dehyde‐3‐phosphate dehydrogenase (*GAPDH*), polyubiquitin, elongation factor 1‐α (*EF‐1α*) and Tubulin (*TUB*), are frequently used as reference genes. However, more and more studies have indicated that the expression patterns of these housekeeping genes are not always stable in different tissues, developmental stages or experimental conditions [8, 9, 10]. Therefore, it is necessary to select optimal reference genes and verify their expression stability before qRT‐PCR experiments.

As costs have gone down, the high‐throughput RNA sequencing (RNA‐seq) has become an important method in molecular biology research for nonmodel species. Screening and identification of reference genes from RNA‐seq data is the most common, economical and efficient method. In RNA‐seq experiments, unigenes with stable expression patterns are selected as candidate reference genes and subjected to qRT‐PCR detection; then they are evaluated by statistical algorithms, such as genorm, normfinder, bestkeeper and deltact, to identify the suitable reference genes [11, 12, 13, 14]. A series of suitable reference genes from fleshy fruits, including blueberry [15, 16], plum [17], peach [18], pepper [19] and kiwifruit [20], have been successfully identified using this strategy.

In recent years, red pitaya has been widely cultivated in southern and southwestern China. Red pitaya fruit is highly attractive and rich in nutritional compounds, such as soluble sugars, organic acid, amino acids, vitamin, betalains, polyphenols and flavonoids [21, 22, 23]. To study the molecular mechanism of red pitaya fruit ripening, we carried out RNA‐seq analysis to identify differentially expressed unigenes, as well as stably expressed unigenes. The latter is a good resource of potential reference genes for normalizing target gene expression.

In this study, based on RNA‐seq analysis, several appropriate genes were selected as candidate reference genes. During fruit ripening of red pitaya, the expression stability of these candidate reference genes was evaluated comprehensively. In addition, soluble sugar accumulation‐related sucrose synthase gene (*SUSY*) and betalains biosynthesis‐related 4, 5‐dihydroxyphenylalanine‐extradiol‐dioxygenase (*DOD* gene) were selected to confirm the accuracy and reliability of the identified stable reference genes. Validated reference genes in this study will be helpful for the future study of the molecular mechanism of red pitaya fruit ripening.

## Materials and methods

### Plant materials

The red pitaya cultivar ‘Zihonglong’ was used in this study; it was collected from the orchard of tropical fruit plantation of Guizhou Academy of Agricultural Sciences, Zhengning County, Anshun City, Guizhou Province, China. Due to its commercial values and resistance to low temperature in winter, the cultivar ‘Zihonglong’ is widely grown and occupies >70% of the total pitaya planting area. This cultivar is also commonly used as experimental materials in studies [23, 24, 25].

Fruit samples were collected from 10 similar healthy plants. According to previous studies, fruit development, seed formation, nutritional quality formation and fruit ripening occurred 1–30 days after artificial pollination (1–30 DAAP) [22, 23, 24]. The number of DAAP, the fruit pulp and peel color, total soluble solids and occurrence of cracking are the main harvest indexes for commercial harvesting [26]. The fruit development of red pitaya was divided into four stages: stage 1 (S1, the initial exponential growth phase 1–14 DAAP), stage 2 (S2, seed formation in 15–19 DAAP), stage 3 (S3, the second exponential growth phase in 20–27 DAAP) and stage 4 (S4, fruit ripening in 28–30 DAAP) [22, 23, and our unpublished research]. In this study, fruit samples were collected at S3 and S4 (20, 23, 25, 27 and 30 DAAP, respectively). The following physiological changes occurred at these five time points: the fruit weight and size increased rapidly (20 DAAP); betalains began to accumulate and the fruit pulp turned pink (23 DAAP); betalains accumulated rapidly, soluble sugars began to accumulate, starch rapidly degraded and the pulp turned red (25 DAAP); the fruit weight and size increased slowly, soluble sugars accumulated rapidly, betalains accumulated rapidly and the pulp turned purplish red (27 DAAP); and soluble sugars and betalains contents reached the highest levels, and the fruit was ripe and ready to be harvested (30 DAAP) [22, 23]. At each sampling time point, we collected nine fruit samples and divided them into three biological replicates [9, 10, 17, 18, 19, 20]; thus, each replicate included three fruit samples. The three biological replicates were collected from three harvest seasons (July, September and October 2017), respectively. The fruit pulp was sliced and quickly frozen in liquid nitrogen immediately after being harvested. All samples were kept at −80 °C for further experiments.

### Total RNA isolation and cDNA synthesis

Total RNA was isolated according to the TRIzol reagent instruction (Invitrogen, Waltham, MA, USA) [25, 27]. The purity and concentration were measured using a NanoDrop 1000 spectrophotometer (Thermo Scientific, Waltham, MA, USA). RNA samples with 1.8 < *A*_260/280_ ratios < 2.2 and *A*_260/230_ ratios > 2.0 were confirmed as qualified samples. The integrity of total RNA samples was detected in 1.2% gel electrophoresis. For each sample, total RNA (1.0 μg) was inversely transcribed for the first‐strand cDNA synthesis using the RevertAid First Strand cDNA Synthesis Kit (Thermo Scientific). To remove genomic DNA, we treated each sample using RNase‐free DNase I (Thermo Scientific). The cDNAs was diluted 10 times as qRT‐PCR templates and stored at −20 °C.

### RNA‐seq data

After total RNA extraction, mRNA isolation, library construction and sequencing [28], a total of 148 Gb high‐quality clean reads were obtained using Illumina HiSeq™ 4000. Sequence assembly and annotation and gene digital expression were analyzed according to Hua *et al*. [28]. After *de novo* assembly, 63 958 unigenes were obtained. The unigenes were then annotated against the National Center for Biotechnology Information nonredundant protein database, Protein family, Kyoto Encyclopedia of Genes and Genomes database, Clusters of Orthologous Groups of proteins database and Gene Ontology database. The clean reads from each sample were mapped to the obtained unigenes, and the fragments per kilobase of exon per million reads mapped (FPKM) value of each unigene was calculated.

### Selection of candidate reference genes and primer design

The selection of candidate reference genes was mainly based on studies on fruits of horticultural crops in recent years, such as grape [8], litchi [9], navel orange [10], plum [17], peach [18], apple [29], watermelon [30], Chinese jujube [31] and cherry [32]. According to the findings of these studies, we selected *ACT7*, *EF‐1α*, eukaryotic initiation factor 4‐α (*IF‐4α*), Polypyrimidine tract‐binding protein‐like (*PTBP*), serine/threonine protein phosphatase 2A (*PP2A*), Elongation factor 2 (*EF2*), glyceraldehyde‐3‐phosphate dehydrogenase (*GAPDH*), Chaperone protein dnaJ 49 (*DNAJ*), Tubulin beta‐8 chain‐like (*TUB*) and Cyclophilin (*CYP*) as candidate reference genes. Some reference genes, including *PTBP* and *DNAJ*, in addition to being reference genes in fruit ripening, were also found to be expressed stably under biotic and abiotic stresses [33, 34, 35]. Therefore, we also randomly selected Heat shock protein 70 (*Hsp70*) that stably expressed under biotic and abiotic stresses [35] and evaluated its expression stability in fruit ripening of red pitaya.

For each selected unigene, the mean values of FPKM, standard deviation (SD) values and coefficient of variation (CV) values of 15 samples were calculated. Then, according to similar studies on pepper [19], kiwifruit [20] and *Reaumuria soongorica* [36], unigenes with the lowest CV value, the FPKM value > 10 and the length > 600 bp were selected as candidate reference gene (Data S1). Selected unigenes were further retrieved against the National Center for Biotechnology Information database using BLASTx to ensure the reliability of annotation results.

According to the selected unigene sequences, specific primer sets for qRT‐PCR were designed using the primer premier 5.0 software (San Francisco, CA, USA) (Table 1). The criteria of primer design were modified from similar studies as follows: melting temperature of 55–62 °C, GC content of 45–65%, primer length of 20–22 bp and amplicon length of 100–150 bp [8, 15, 36, 37, 38]. To validate the correct amplification, the amplicons of each primer set were detected on 1.5% agarose gel electrophoresis and subjected to sequencing [10, 36]. Melting‐curve analysis for each primer set was conducted to further confirm the specificity of PCR amplification. To calculate the PCR amplification efficiency of each primer set, series of 10‐fold dilution of cDNA, as well as ddH_2_O, were used as templates for qRT‐PCR. The standard curve was generated for the calculation of amplification efficiency (*E*) and correlation coefficients (*R* ^2^) of each primer set.

**Table 1:** Primer sets of 11 candidate reference genes for red pitaya fruits.

Gene	Primer sequence (5′–3′)	Size (bp)	*E*/%	*R* ^2^
*ACT7*	GGTCCTCTTCCAGCCTTCAT	133	104.08	0.9994
	TGTACCACCACTGAGCACAA			
*EF‐1α*	GCTGTCAAGGATCTCAAGCG	149	93.85	0.9963
	GTGTGGCAATCAAGGACTGG			
*IF‐4α*	AATTCAGCAAGCCCAGTCAG	116	102.35	0.9996
	CTCTGGTAGGAGCGAGAACA			
*PTBP*	AGCGTCAAATTCCAAAGCCA	105	111.20	0.9971
	ATCCAAGCCCACACCTAACT			
*PP2A*	TCAGCTGTGTTCCCTACCAG	109	102.82	0.9959
	ACAGGTTCAACTTGCTCAGG			
*EF2*	TGGTGCTGGAGAACTTCACT	145	95.22	0.9928
	CTTGCTCATCACAGTACGGC			
*Hsp70*	CTGGCCTATGAGAGTGCAGA	119	99.93	1.0000
	GCAGAGAGATTCCGGGATGA			
*GAPDH*	AGGAAGGACTGGAGAGGAGG	100	115.20	0.9980
	CATTGAGGTCCGGCAAAACT			
*DNAJ*	TACTTGCCCTTCTCAGAGCC	119	98.22	0.9983
	CTGCGATCAAAGTCCAGTGA			
*TUB*	CCTTGACTGTGCCTGAGTTG	145	106.71	0.9994
	ATCATCTGCTCGTCCACCTC			
*CYP*	CTAACACCAACGGATCGCAG	107	107.16	0.9954
	CTTCACCACATCCATCCCCT			

### qRT‐PCR analysis

The qRT‐PCR was performed in the CFX Connect™ Real‐Time PCR detection system (Bio‐Rad, Hercules, CA, USA). The amplification mixture contained 1.0 μL of cDNA template, 1.6 μL of primer pairs, 10.0 μL of Power SYBR Green PCR Master Mix (Applied Biosystems, Foster City, CA, USA) and 7.4 μL of ddH_2_O. The amplification program was set according to previous studies as follows: 95 °C for 3 min, 40 cycles of 95 °C for 10 s, 55 °C for 20 s and then 72 °C for 20 s [30, 37]. Four technical replicates were conducted for each reaction.

### Statistical analysis

Four common software packages, including genorm, normfinder, bestkeeper and deltact, were applied to the evaluation of expression stability of candidate reference genes [11, 12, 13, 14]. For the data input of genorm and normfinder analysis, cycle threshold (Ct) values were transformed to relative expression values by the formula of 2^(−ΔCt)^ (ΔCt = Ct value for each gene − minimum Ct value for each gene in all samples). The genorm software calculated the gene stability *M* value to determine the expression stability of candidate reference genes. The *M* value represented the average degree of the pairwise variation of a candidate reference gene compared with other genes. As a result, the candidate reference gene with the lowest *M* value had the highest expression stability. In addition, the genorm software could calculate the pairwise variation *V_n_*/*V_n _*_+ 1_ value to determine the optimal number of reference genes for accurate normalization. When the *V_n_*/*V_n_*_ + 1_ value was less than the cutoff value of 0.15, no more reference gene was required [11]. The normfinder software calculated the intragroup and intergroup variation values and provided the stability *M* value for each candidate reference gene. The candidate gene with the lowest stability *M* value was considered as the most stable reference gene [12].

The bestkeeper software estimated the expression stability based on the SD and CV values of Ct values for each candidate reference gene. The lowest SD and CV value suggested the highest expression stability [13]. The deltact software calculated the relative expression of all combinations of gene pairs in each sample. The expression stability was measured by the variation of gene expression differences between samples. The most stable candidate had the lowest CV and SD value [14]. Finally, the comprehensive ranking of the expression stability of candidate reference genes was analyzed using reffinder software (https://www.heartcure.com.au/reffinder/). The lowest ranking value indicated the highest expression stability.

### Normalization of the red pitaya *SUSY* and *DOD* gene expression

The SUSY is the key enzyme for sucrose synthesis and degradation, which plays critical roles in carbon partitioning and soluble sugar accumulation in fleshy fruits [39, 40]. In the biosynthetic pathway of betalains, the DOD is a key enzyme that catalyzes the formation of 4, 5‐seco‐dihydroxyphenylalanine from l‐ dihydroxyphenylalanine [28, 41]. From the RNA‐seq result for red pitaya fruits, the significantly differentially expressed *SUSY*‐related and *DOD*‐related unigenes with the CV values of 0.75 and 0.84, respectively, were used as target genes (Data S1). The BLASTx analysis suggested that the *SUSY*‐related and *DOD*‐related unigenes showed amino acid sequence identity of 94% to *Beta vulgaris* SUSY isoform X2 (GenBank: XP_019106894) and 97% to *Carnegiea gigantea* DOD (GenBank: MN136222), respectively. Two primer sets (Forward: 5′‐TAACCCGTTTGCTCCCTGAT‐3′/Reverse: 5′‐AACAATGCCCTTTTCGGTCC‐3′ for *SUSY*; Forward: 5′‐AGCTTCTCTTGTACCATCTTTCT‐3′/Reverse: 5′‐AATTTAGCTTGAACTTGATGCCA‐3′ for *DOD*) were designed for the qRT‐PCR detection. Gene expression levels during fruit ripening were calculated using the most stable and unstable reference genes from this study and *β‐ACT* in a previous study [28]. After qRT‐PCR detection, calculations of significant differences between qRT‐PCR results by various candidate genes and correlation analysis with the RNA‐seq data were conducted using Microsoft Excel (v. 2007).

## Results

### Selection of candidate reference genes from RNA‐seq results

**Fig. 1:**
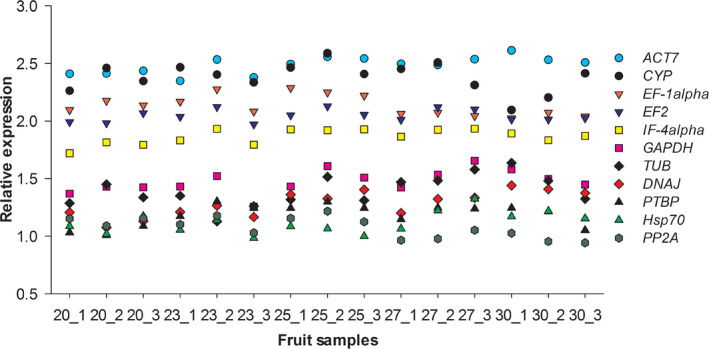
Relative expression patterns of 11 candidate reference genes across fruit samples of red pitaya, based on the RNA‐seq data. The relative expression level of each candidate was represented by the log_10_ (FPKM) value. The FPKM was calculated from the RNA‐seq data.

According to the types of reference gene with stable expression patterns in fleshy fruits [8, 9, 10, 17, 18, 29, 30, 31, 32], we screened candidate reference genes from the RNA‐seq data during fruit ripening of red pitaya. In total, we obtained 11 types of candidate reference gene, as well as their digital expression levels. These candidate reference genes contained *ACT7*, *EF‐1α*, *IF‐4α*, *PTBP*, *PP2A*, *EF2*, *GAPDH*, *Hsp70*, *DNAJ*, *TUB* and *CYP*. In all fruit samples, the average FPKM values of 11 candidate reference genes ranged from 12.10 to 309.75 (Table 2). The expression pattern of each candidate reference gene was relatively stable and did not exhibit obvious changes with fruit ripening (Fig. 1). The CV values of FPKM of each candidate reference gene were 0.14–0.31, which were consistent with the criteria for screening candidate reference genes from RNA‐seq data in pepper and kiwifruit [19, 20].

**Table 2:** Candidate reference genes from RNA‐seq experiment for red pitaya fruits.

Gene	Description	FPKM value		
		Mean	SD	CV
*ACT7*	Actin‐7	309.75	51.22	0.17
*CYP*	Cyclophilin	249.42	66.84	0.27
*EF‐1α*	Elongation factor 1‐alpha	139.05	30.01	0.22
*EF2*	Elongation factor 2	111.98	13.67	0.12
*IF‐4α*	Eukaryotic initiation factor 4A‐9	74.14	10.64	0.14
*GAPDH*	Glyceraldehyde‐3‐phosphate dehydrogenase	30.55	6.98	0.23
*TUB*	Tubulin beta‐8 chain	25.97	8.10	0.31
*DNAJ*	Chaperone protein dnaJ 49	19.69	4.84	0.25
*PTBP*	Polypyrimidine tract‐binding protein	15.65	3.23	0.21
*Hsp70*	Heat shock 70 kDa protein 17	13.39	3.09	0.23
*PP2A*	Serine/threonine protein phosphatase 2A	12.10	2.46	0.20

Meanwhile, a reference gene *β‐ACT* used in previous studies to normalize target gene expression was also evaluated, together with 11 candidate reference genes [28, 42].

### Specificity and efficiency of PCR amplification

After RT‐PCR amplification using cDNA as templates, the amplicons of expected length were detected by agarose gel electrophoresis and sequenced. In the melting curve analysis, a single peak for each primer set further revealed the specificity of PCR amplification (Fig. S1). The ddH_2_O was used as the PCR template, and no bands or obvious amplification curves were observed. To calculate the amplification efficiency, we conducted the standard curve analysis through qRT‐PCR amplification in a dilution series of cDNA template or ddH_2_O. The amplification efficiency of 11 primer sets varied from 93.85% to 115.20%, and the correlation coefficients (*R* ^2^ values) ranged from 0.9928 to 1.000 (Table 1). These results suggested that 11 primer sets had high amplification specificity and efficiency.

### Expression patterns of candidate reference genes

**Fig. 2:**
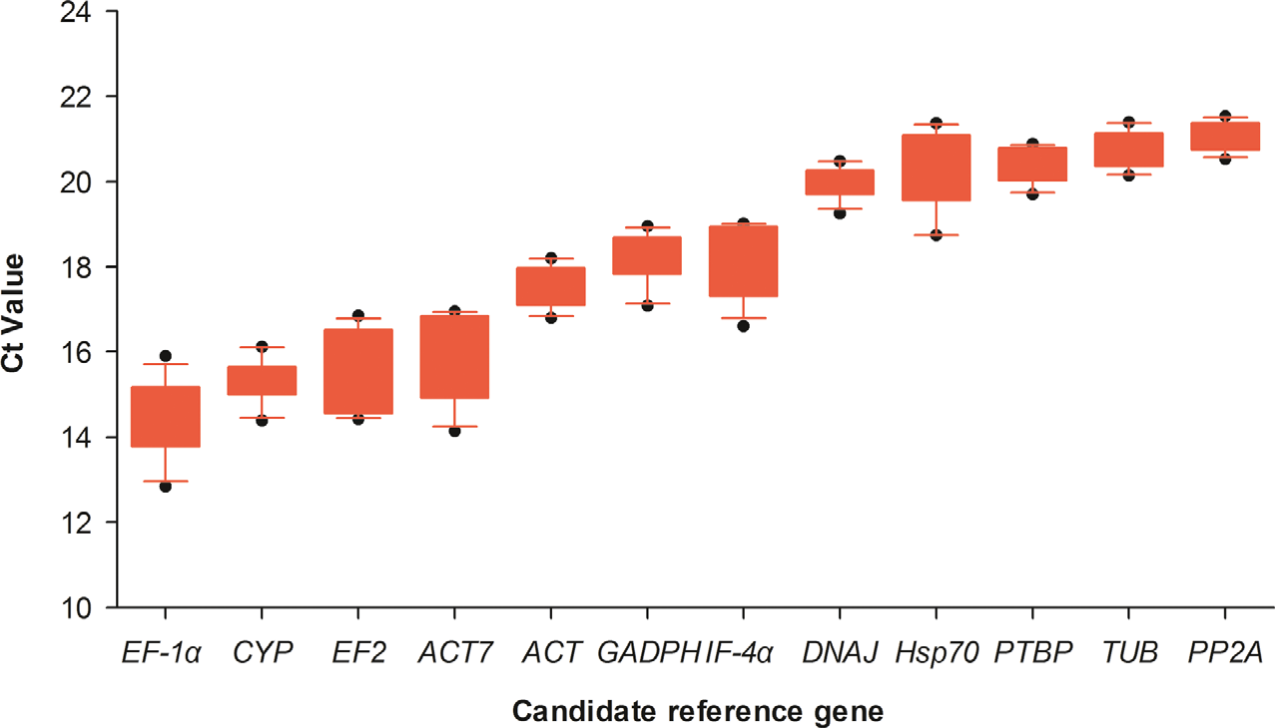
Ct values distribution of 12 candidate reference genes across fruit samples of red pitaya. Box represented the 1/4 and 3/4 quartiles, and whisker caps represented 10/90 percentiles. Lines across boxes showed the median values, and dots indicated the outliers of 10th/90th percentiles.

After qRT‐PCR amplification, the Ct values of 12 candidate reference genes were obtained from the 15 fruit samples. As shown in Fig. 2, the Ct values of these candidate reference genes varied from 12.838 to 22.508. *EF‐1α*, *CYP*, *ACT7* and *EF2* showed relatively low median Ct values (14.577–15.699), of which *EF‐1α* had the highest expression level. The median Ct values of *IF‐4α*, *GAPDH* and *β‐ACT* ranged from 17.778 to 18.327. *PTBP*, *PP2A*, *Hsp70*, *DNAJ* and *TUB* displayed relatively high median Ct values (20.167–21.267), of which *PP2A* exhibited the lowest expression level.

### Expression stability analysis

To obtain reliable reference genes, we evaluated the expression stability of 12 candidate reference genes using four popular software packages: genorm, normfinder, bestkeeper and deltact. Finally, the overall result was comprehensively evaluated by the reffinder software.

**Fig. 3:**
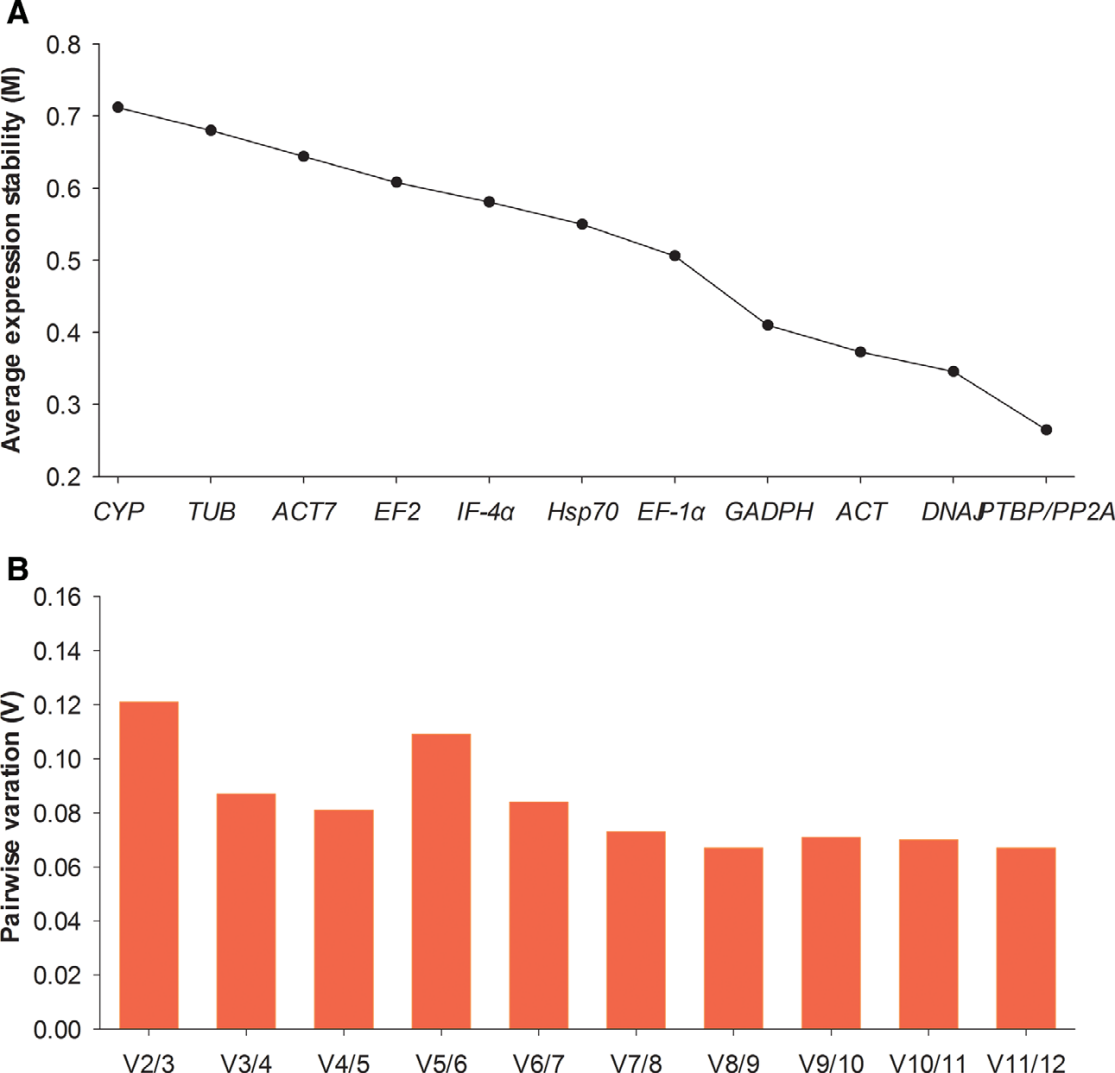
Expression stability (A) and pairwise variation (B) analysis of 12 candidate reference genes calculated by genorm software. The lower *M* value (average expression stability) indicated the higher expression stability.

The genorm software was the most common software for reference gene analysis, and it determined the expression stability by expression stability values (*M* values). The lower *M* value indicated the higher expression stability of the reference gene, with a cutoff *M* value of 1.5 [11]. As shown in Fig. 3A, all *M* values of 12 candidate reference genes were <1.5 and were sorted as follows: *PTBP* = *PP2A* < *DNAJ* < *β‐ACT* < *GAPDH* < *EF‐1α* < *Hsp70* < *IF‐4α* < *EF2* < *ACT7* < *TUB* < *CYP*. This result suggested that *PTBP* and *PP2A* were the two most stable reference genes, whereas *TUB* and *CYP* were the top two unstable ones.

Meanwhile, the optimal number of reference genes was also determined by the pairwise variation value (*V_n_*_/_ *_n_*_+1_) calculated from the genorm software. The *V_n_*_/_ *_n_*_+1_ value should be lower than 0.15 to meet the experimental requirement [11]. Based on the pairwise variation analysis (Fig. 3B), the *V*_2/3_ value (0.121) was first lower than the cutoff value of 0.15. It indicated that only the two most stable reference genes were necessary for normalization of target gene expression.

The data input and analysis using the normfinder software were similar to the genorm software. The lower *M* value indicated the higher expression stability of the reference gene [12]. The result was shown in Table 3; the *M* value of *GADPH* was the lowest, followed by *PTBP*, *DNAJ*, *β‐ACT* and *PP2A*. *ACT7* presented the highest *M* value. Therefore, *GADPH* was the most stable reference gene, whereas *ACT7* was the most unstable one.

**Table 3:** Expression stability ranking of 12 candidate reference genes calculated by normfinder, bestkeeper and deltact software packages.

Rank	normfinder		bestkeeper		deltact	
	Gene	*M*	Gene	SD	Gene	SD
1	*GADPH*	0.287	*DNAJ*	0.315	*GADPH*	0.611
2	*PTBP*	0.357	*β‐ACT*	0.387	*PTBP*	0.618
3	*DNAJ*	0.357	*PP2A*	0.427	*DNAJ*	0.621
4	*β‐ACT*	0.398	*PTBP*	0.439	*PP2A*	0.644
5	*PP2A*	0.422	*TUB*	0.463	*β‐ACT*	0.648
6	*EF‐1α*	0.449	*GADPH*	0.474	*EF‐1α*	0.672
7	*Hsp70*	0.501	*EF‐1α*	0.595	*Hsp70*	0.696
8	*IF‐4α*	0.530	*IF‐4α*	0.607	*IF‐4α*	0.711
9	*EF2*	0.583	*CYP*	0.637	*EF2*	0.760
10	*TUB*	0.700	*Hsp70*	0.656	*TUB*	0.822
11	*CYP*	0.784	*EF2*	0.704	*ACT7*	0.890
12	*ACT7*	0.800	*ACT7*	0.853	*CYP*	0.895

The bestkeeper software evaluated the expression stability of candidate reference genes by calculating the SD and CV of Ct values. The lower value of SD and CV indicated the higher expression stability [13]. As listed in Table 3, the SD value of *DNAJ* was the lowest, followed by *β‐ACT*, *PP2A*, *PTBP*, *TUB* and *GADPH*. The SD value of *ACT7* was the highest among 12 candidate reference genes. The ranking suggested that *DNAJ* showed the highest expression stability, whereas *ACT7* was the lowest one (Table 3).

The DeltaCt method also measured the gene expression stability by calculating the SD and CV values of Ct values. The lower value of SD and CV suggested the higher expression stability [14]. As shown in Table 3, *GADPH* with the lowest SD value exhibited the highest expression stability, and *CYP* with the highest SD value was the most unstable one.

The expression stability of 12 candidate reference genes showed minor differences when using different statistical methods. The RefFinder was a web‐based evaluation tool, which comprehensively ranked the results of the earlier four methods and generated a consensus result. According to the ranking from four statistical methods, the geometric mean value of each reference gene was calculated. Similarly, the lower ranking value indicated the higher expression stability. The result was listed in Table 4; *PTBP* had the lowest ranking value, followed by *DNAJ*, *GADPH*, *PP2A*, *β‐ACT*, *EF‐1α*, *Hsp70*, *IF‐4α*, *TUB*, *EF2* and *CYP*, and *ACT7* had the highest ranking value. Therefore, *PTBP* and *DNAJ* were the top two suitable reference genes with the high expression stability during fruit ripening of red pitaya, whereas *ACT7* and *CYP* were the top two unstable ones.

**Table 4:** RefFinder ranking of 12 candidate reference genes.

Rank	Candidate genes	Ranking value
1	*PTBP*	2.00
2	*DNAJ*	2.28
3	*GADPH*	2.34
4	*PP2A*	2.78
5	*β‐ACT*	3.56
6	*EF‐1α*	6.24
7	*Hsp70*	7.65
8	*IF‐4α*	8.00
9	*TUB*	8.61
10	*EF2*	9.46
11	*CYP*	10.93
12	*ACT7*	11.22

### Normalization of the red pitaya *SUSY* and *DOD* gene expression

**Fig. 4:**
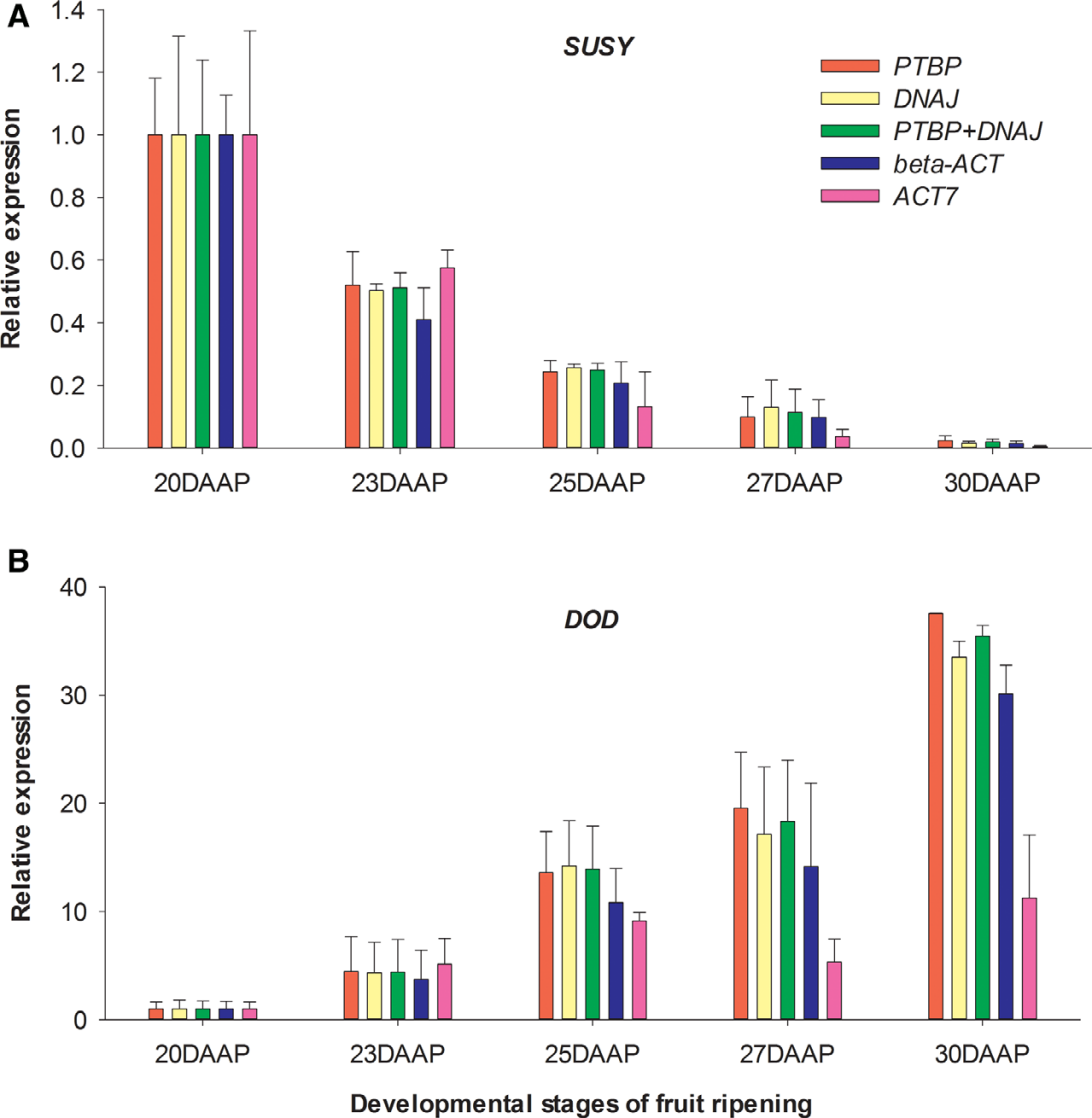
Expression patterns of *SUSY* and *DOD* gene normalizing by different reference genes during fruit ripening of red pitaya. The five developmental stages of fruit ripening were indicated as 20, 23, 25, 27 and 30 DAAP. Error bars showed the mean standard error (*n* = 3).

To test the reliability of the identified stable reference genes, *PTBP*, *DNAJ, β‐ACT* and *ACT7* were used as reference genes to normalize the expression patterns of *SUSY* and *DOD* gene. As shown in Fig. 4, when normalizing by *PTBP*, *DNAJ*, *PTBP* + *DNAJ*, *β‐ACT* or *ACT7*, the *SUSY* expression levels gradually decreased during fruit ripening, which exhibited similar patterns with the RNA‐seq result. Calculated by *ACT7* or *β‐ACT*, the decrease of *SUSY* gene expression was more obvious at 25 and 27 DAAP (by *ACT7*) or at 23 DAAP (by *β‐ACT*), compared with *PTBP*, *DNAJ* or *PTBP* + *DNAJ*. Furthermore, the *R* ^2^ values of *PTBP* (0.98), *PTBP* + *DNAJ* (0.98) and *DNAJ* (0.97) with the RNA‐seq result were a little higher than *β‐ACT* (0.96) and *ACT7* (0.94).

The *DOD* gene expression patterns normalizing by *PTBP*, *DNAJ*, *PTBP* + *DNAJ* or *β‐ACT* were up‐regulated during fruit ripening (Fig. 4), which was also similar to the RNA‐seq result. Nevertheless, at 25, 27 or 30 DAAP, the increase of *DOD* gene expression normalizing by *β‐ACT* was lower than by *PTBP*, *DNAJ* or *PTBP* + *DNAJ*. Similarly, the *R* ^2^ values of *PTBP* (0.79), *DNAJ* (0.82) and *PTBP* + *DNAJ* (0.81) with the RNA‐seq result were obviously higher than *β‐ACT* (0.75). When normalizing by *ACT7*, the expression level of *DOD* decreased at 27 DAAP, which significantly differed from the RNA‐seq result.

## Discussion

The development and ripening of fleshy fruit is accompanied by multiple physiological and biochemical processes, such as degreening, the accumulation of soluble sugar, organic acid and pigments, seed formation and starch degradation [17, 43]. Studying the physiological functions of key genes in fruit development and ripening is of great significance for regulating and improving fruit quality. Suitable reference genes are necessary for the quantification of key genes expression patterns. To date, several studies on identifying suitable reference genes during fruit development and ripening have been conducted. As found in these studies, genes with the most stable expression patterns are always different in different fruit species or at different developmental stages, indicating the complexity of suitable reference genes in fleshy fruits [9, 10, 18, 20, 29]. Therefore, it is necessary to conduct careful verification to identify the most stable reference genes during fruit ripening of red pitaya.

In this study, to identify the most stable reference gene, we used four statistical approaches to estimate the expression stability of 12 candidate reference genes during fruit ripening of red pitaya. The ranking orders generated by genorm, normfinder, bestkeeper and deltact were not completely identical (Fig. 2; Table 3). Other studies also reported different ranking orders using different statistical approaches when identifying suitable reference genes for grapevine [8], litchi [9], navel orange [10] and peach [18] at fruit developmental stages. In our study, although there were differences in the ranking orders of expression stability of candidate reference genes, the rank of the most stable reference genes did not show great differences. Of the 12 candidate reference genes, *PTBP* ranked first, second, fourth and second, calculated by genorm, normfinder, bestkeeper and deltact, respectively. Then after RefFinder analysis, *PTBP* ranked first (Table 4). Similarly, the final overall ranking of *DNAJ* was second. In conclusion, we proposed *PTBP* and *DNAJ* as suitable reference genes for normalization of target gene expression during fruit ripening of red pitaya.

In plants and animals, PTBPs as a family of RNA‐binding proteins are involved in RNA metabolic processes, such as alternative splicing, maintenance of mRNA stability, protein transcription and translation, and polyadenylation [43, 44, 45]. DNAJ proteins are cochaperones of Hsp70s, and they play an essential role together in protein folding, degradation and refolding [46]. In plants, DNAJ proteins are related with biotic and abiotic stress responses [47]. *PTBP* and *DNAJ* display high expression stability in fleshy fruits or stress conditions and can be used as suitable reference genes. In fruit development of kiwifruit [20], watermelon [30] and tomato [48], both *PTBP* and *DNAJ* show stable expression patterns. Under stress conditions, such as heat, drought, cold, oxidative and salt, hormones and heavy metal treatments, *PTBP* from *Lycoris aurea* [33] and *Peucedanum praeruptorum* Dunn [34] and *DNAJ* from *Salix matsudana* [35] are the most stable reference genes for gene normalization. Therefore, in addition to being used as stable reference genes during fruit ripening, the red pitaya *PTBP* and *DNAJ* may be used for gene normalization under stress conditions, which needs further verification.

The *ACT* gene is the most frequently used housekeeping gene, and it is widely used as a reference gene in plants. Nevertheless, past findings have shown that isoforms of *ACT* gene display very different expression stability in different fleshy fruits or at different fruit developmental stages. For example, *ACT* was identified as the most stable reference gene during whole developmental stages of litchi and jujube fruits [9, 31]; however, it was found to be the least stable one during the navel orange fruit ripening [10]. In our study, among the 12 selected candidate reference genes, the expression stability of *ACT7* was found to be the most unstable one during fruit ripening (20, 23, 25, 27 and 30 DAAP). However, in another study, *ACT (1)* was identified to be the most stable gene for seven developmental stages of pitaya fruit (13, 16, 19, 23 25, 27 and 29 DAAP) [37]. Surprisingly, although the primer sets for *ACT7* and *Actin (1)* were both designed from *Hpactin7* (GenBank: MF356257), *ACT7* and *Actin (1)* showed very different expression stability. We speculated that the reason for this was because the fruit samples were collected at different developmental stages. In our study, fruit samples were collected from S3 (20, 23, 25 and 27 DAAP) and S4 (30 DAAP). In the previous study, fruit samples were harvested from late S1 (13 DAAP), S2 (16 and 19 DAAP), S3 (23, 25 and 27 DAAP) and S4 (29 DAAP) [37]. Therefore, the difference in fruit developmental stages led to the discrepancy of the *ACT* expression stability. Before normalization of qRT‐PCR data, it is indeed necessary to carefully evaluate the expression stability of reference genes within specific experimental samples.

To validate the accuracy of most stable reference genes from this study, expression patterns of *SUSY* and *DOD* were evaluated during fruit ripening. *SUSY* and *DOD* expression patterns normalized by *PTBP*, *DNAJ* or *PTBP* + *DNAJ* exhibited high consistency with the RNA‐seq data, which further confirmed that *PTBP* and *DNAJ* were suitable reference genes for the quantification of gene expression levels. Meanwhile, we also evaluated the expression stability of *β‐ACT*, which had been used as the only reference gene to normalize target gene expression in red pitaya fruit [28, 42]. It was worth noting that *β‐ACT* was first isolated from pitaya stem tissues without verifying its expression stability [25]. In this study, the expression stability of *β‐ACT* ranked fifth among 12 candidate reference genes (Table 4). The *SUSY* and *DOD* expression levels normalized by *β‐ACT* showed minor differences with *PTBP*, *DNAJ* or *PTBP* + *DNAJ* and had slightly lower *R* ^2^ values than the RNA‐seq result. In future study, we recommend *PTBP* and *DNAJ* as suitable reference genes during fruit ripening of red pitaya.

## Conclusion

This study evaluated the suitable reference genes during fruit ripening of red pitaya. The overall results reveal that *PTBP* and *DNAJ* are suitable reference genes during fruit ripening of red pitaya. The present results will facilitate the quantification of target gene expression patterns by qRT‐PCR detection and will provide a foundation for the study of molecular biological mechanisms of red pitaya fruit ripening.

## Conflict of interest

The authors declare no conflict of interest.

## Author contributions

QZ designed the overall research, collected the fruit samples and wrote the manuscript. XW collected the fruit samples and conducted the experiments. YQ contributed analytical tools and analyzed data. YM designed the overall research and edited the manuscript.

## Supporting information


**Fig. S1.** Melting curves for 11 candidate reference genes in 15 fruit samples.

**Data S1.** The sequences of 11 candidate reference genes and 2 target genes (*SUSY* and *DOD*) from RNA‐seq for red pitaya fruits.

## Data Availability

The sequences of 11 candidate reference genes and 2 target genes (*SUSY* and *DOD*) from RNA‐seq analysis for red pitaya fruits are listed in Data S1.
