# Supplementary material for: Selection and validation of reference genes for qRT‐PCR analysis during fruit ripening of red pitaya (Hylocereus polyrhizus)
Source: FEBS Open Bio. 2021 Oct 6;11(11):3142–52. doi: 10.1002/2211-5463.13053 (PMC8564333; doi:10.1002/2211-5463.13053)

**Supplementary Figure S1.** Melting curves for 11 candidate reference genes in 15 fruit samples

***ACT7***


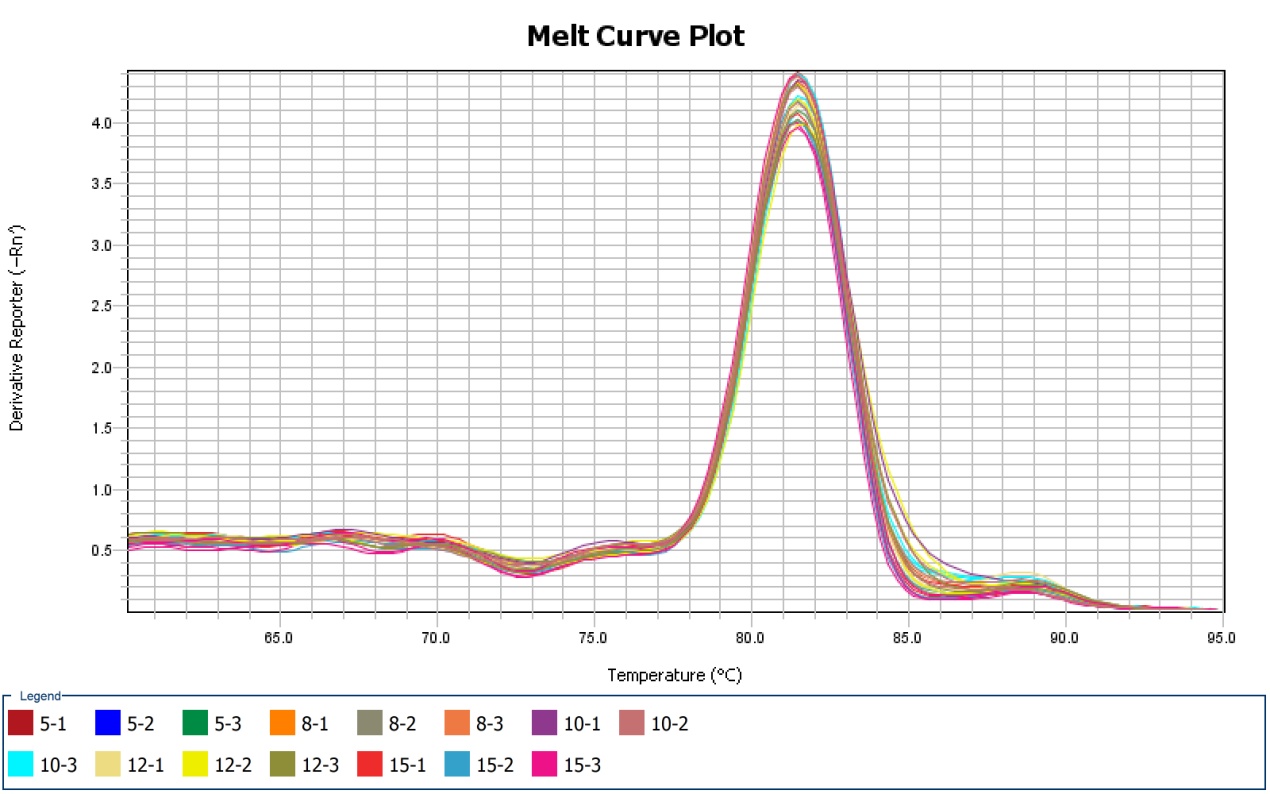


***CYP***


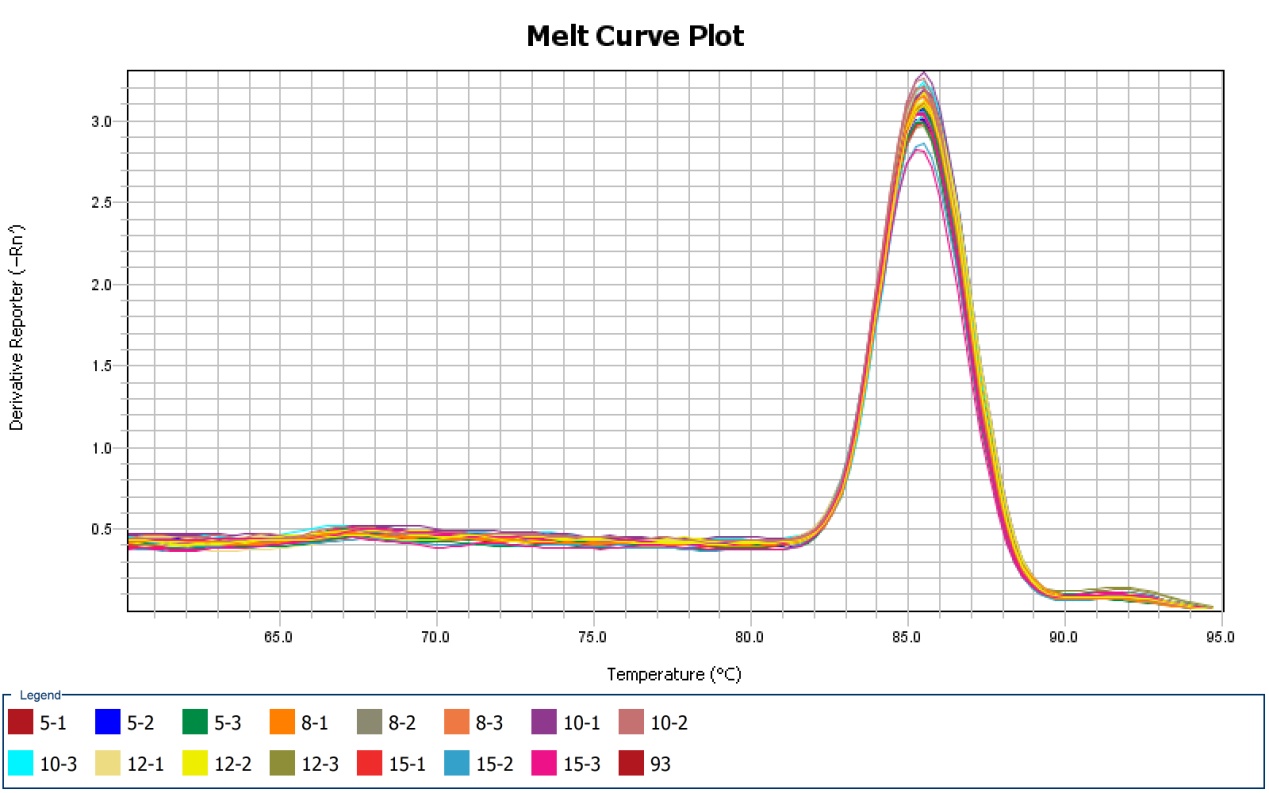


***EF-1α***

**
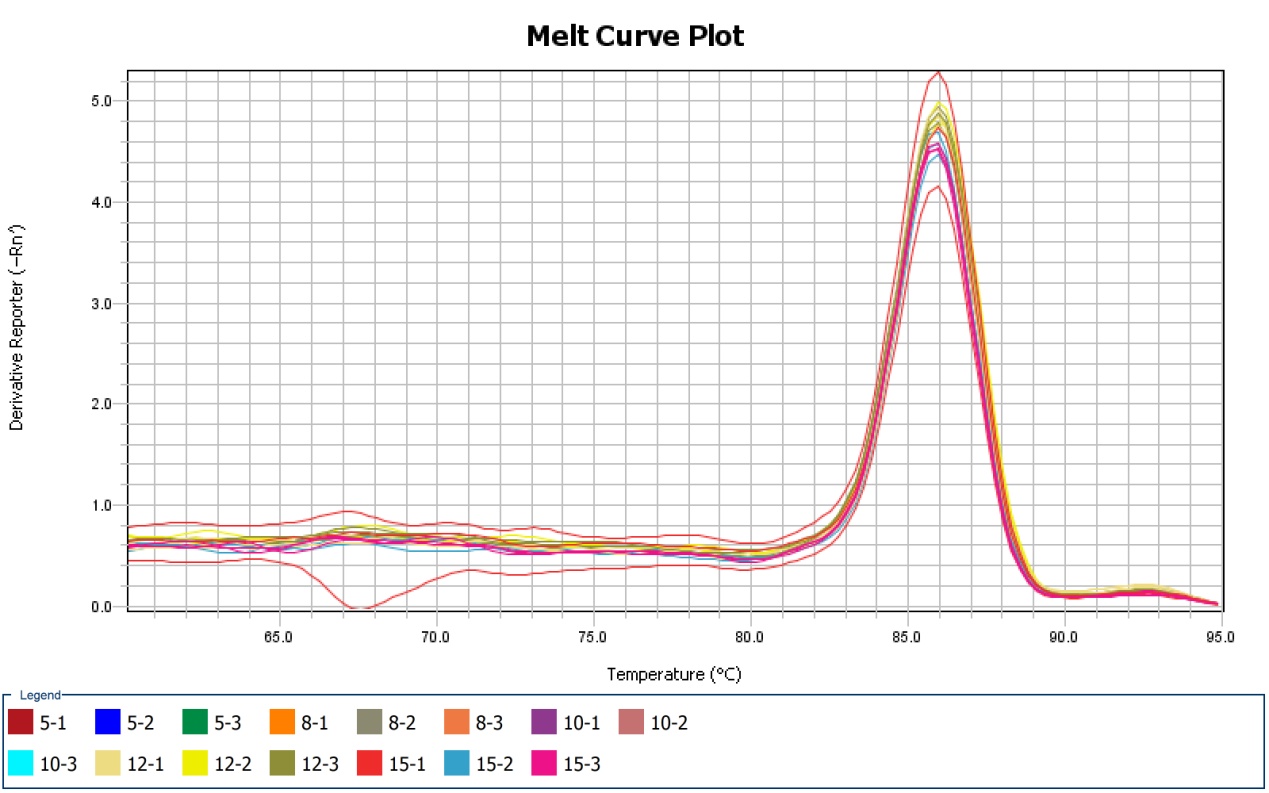
**

***EF2***

**
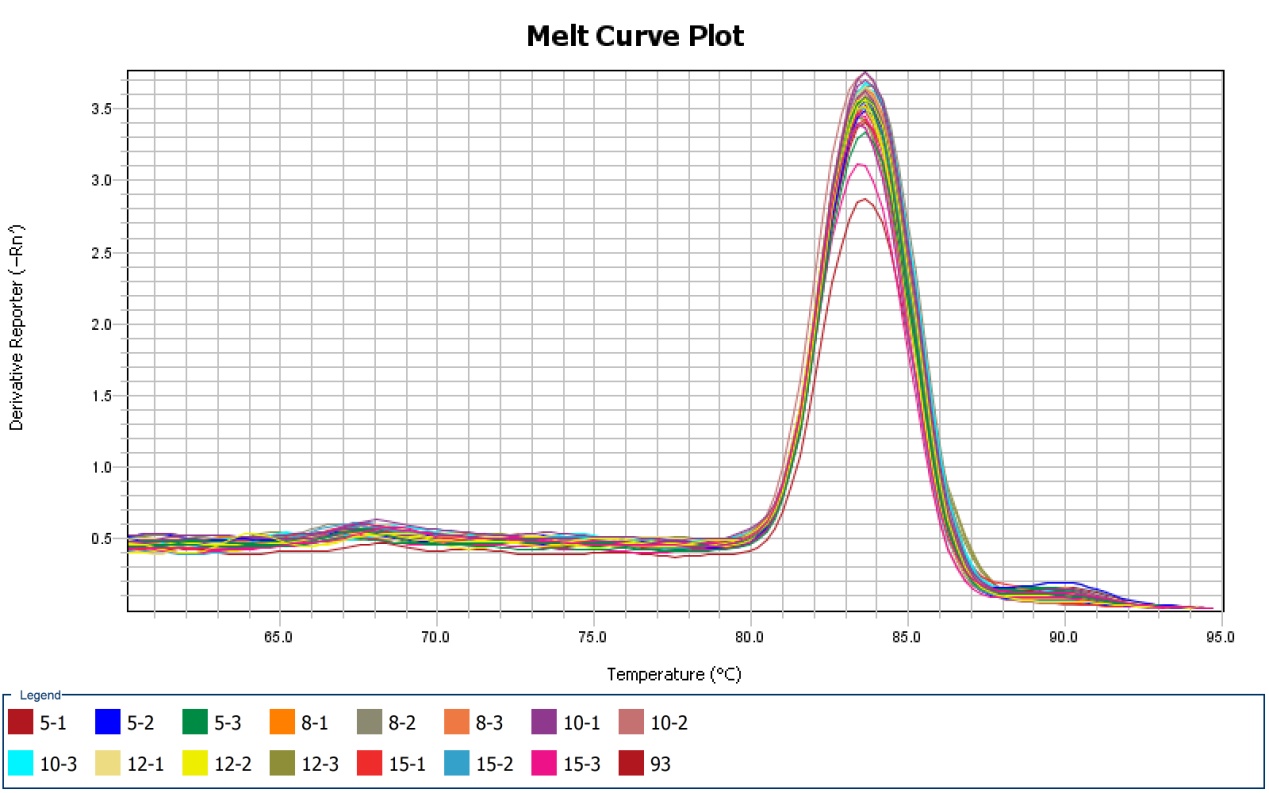
**

***IF-4α***

**
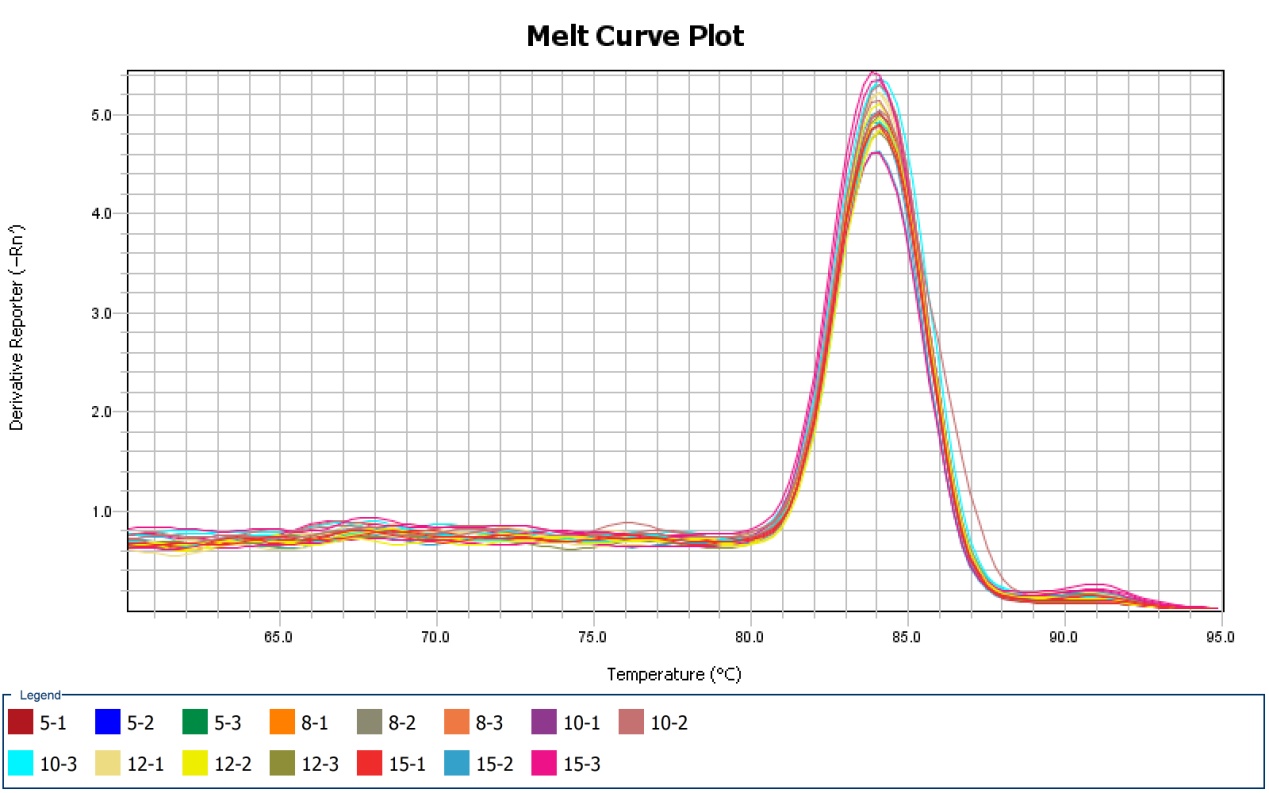
**

***GAPDH***

**
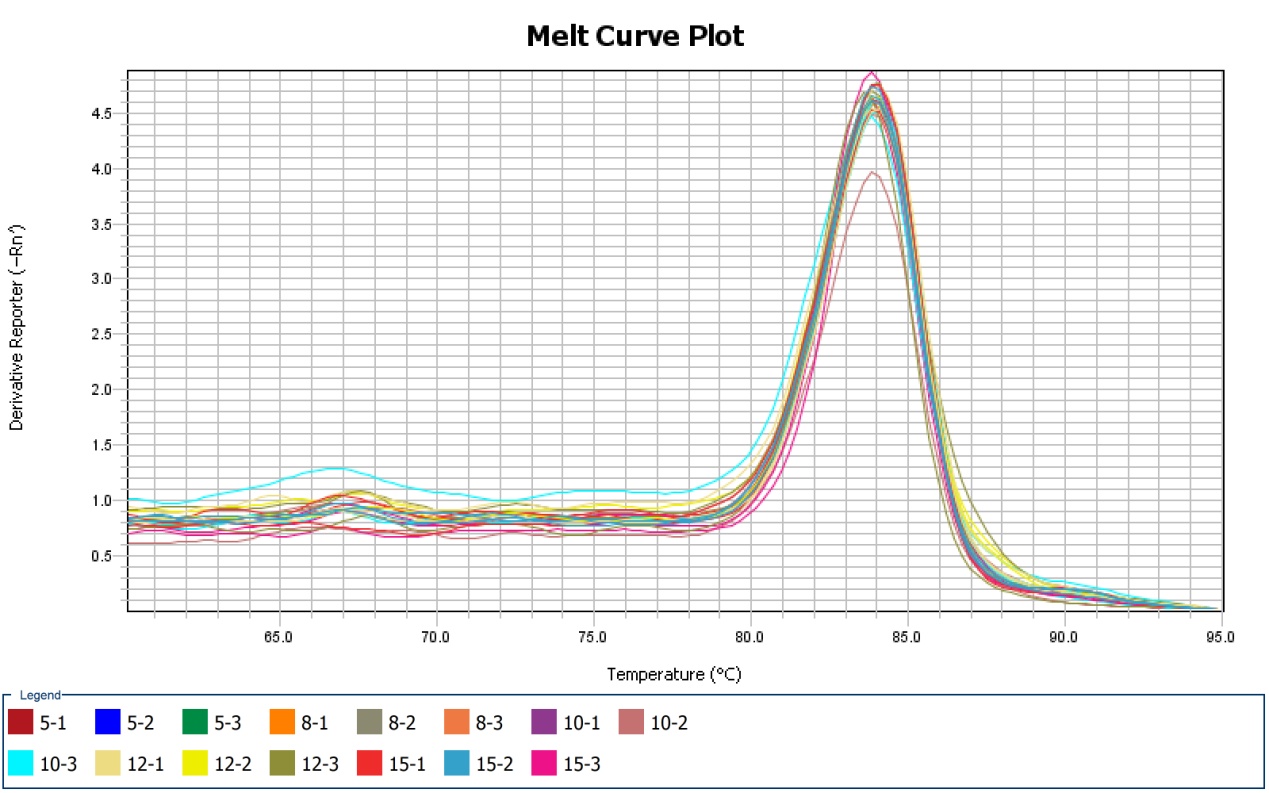
**

***TUB***

**
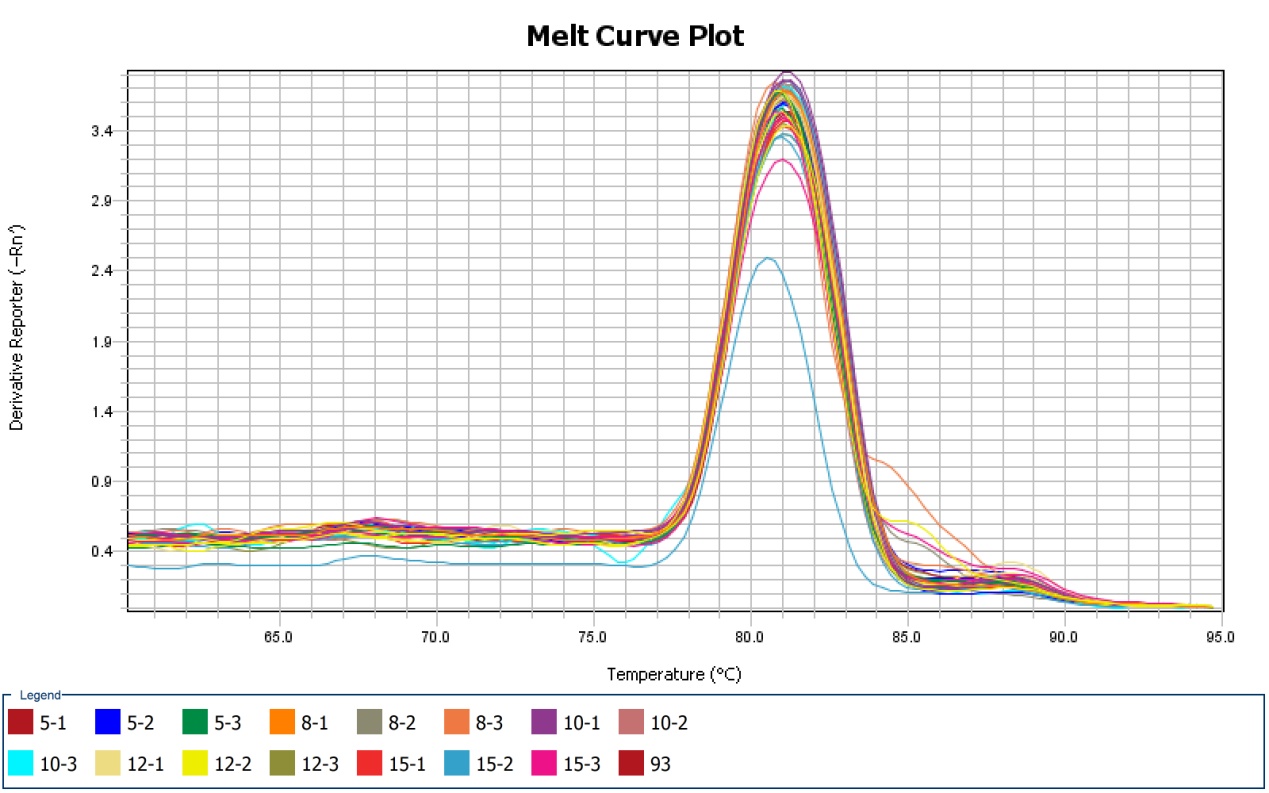
**

***DNAJ***

**
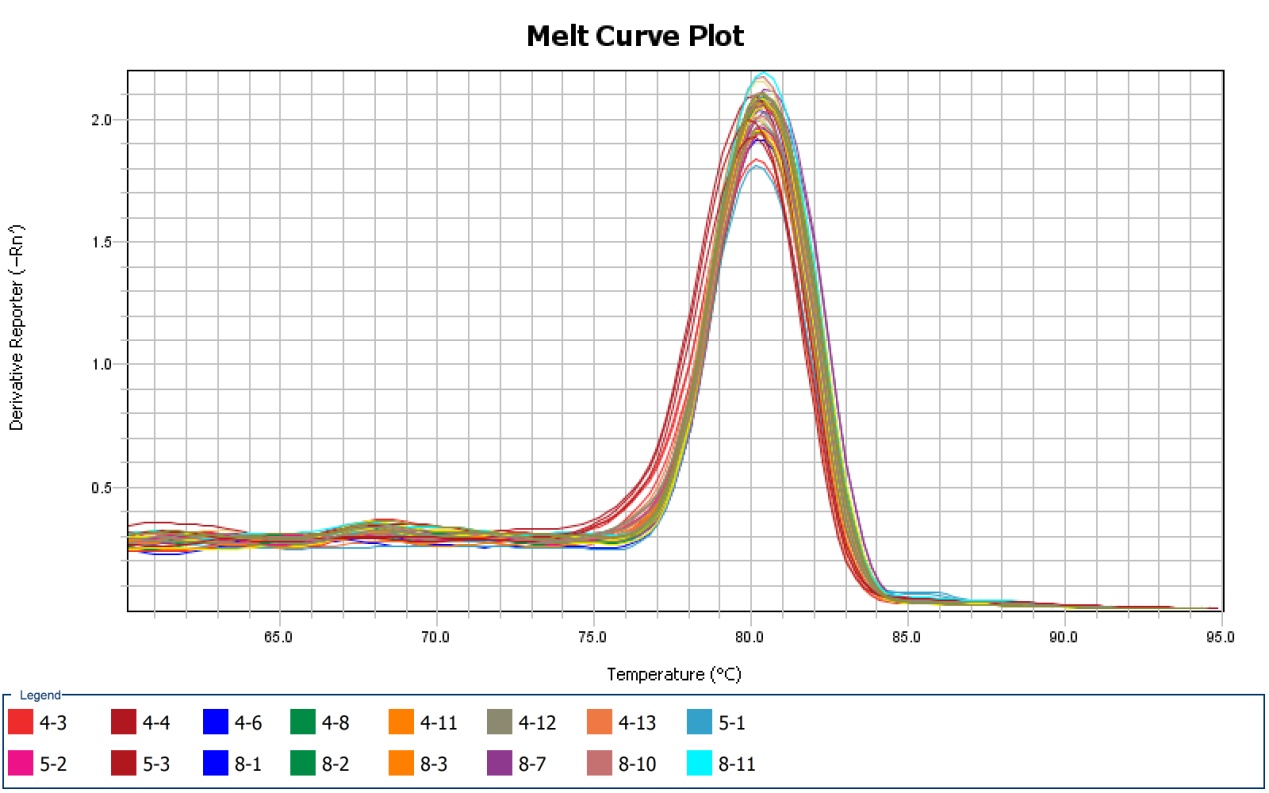
**

***PTBP***

**
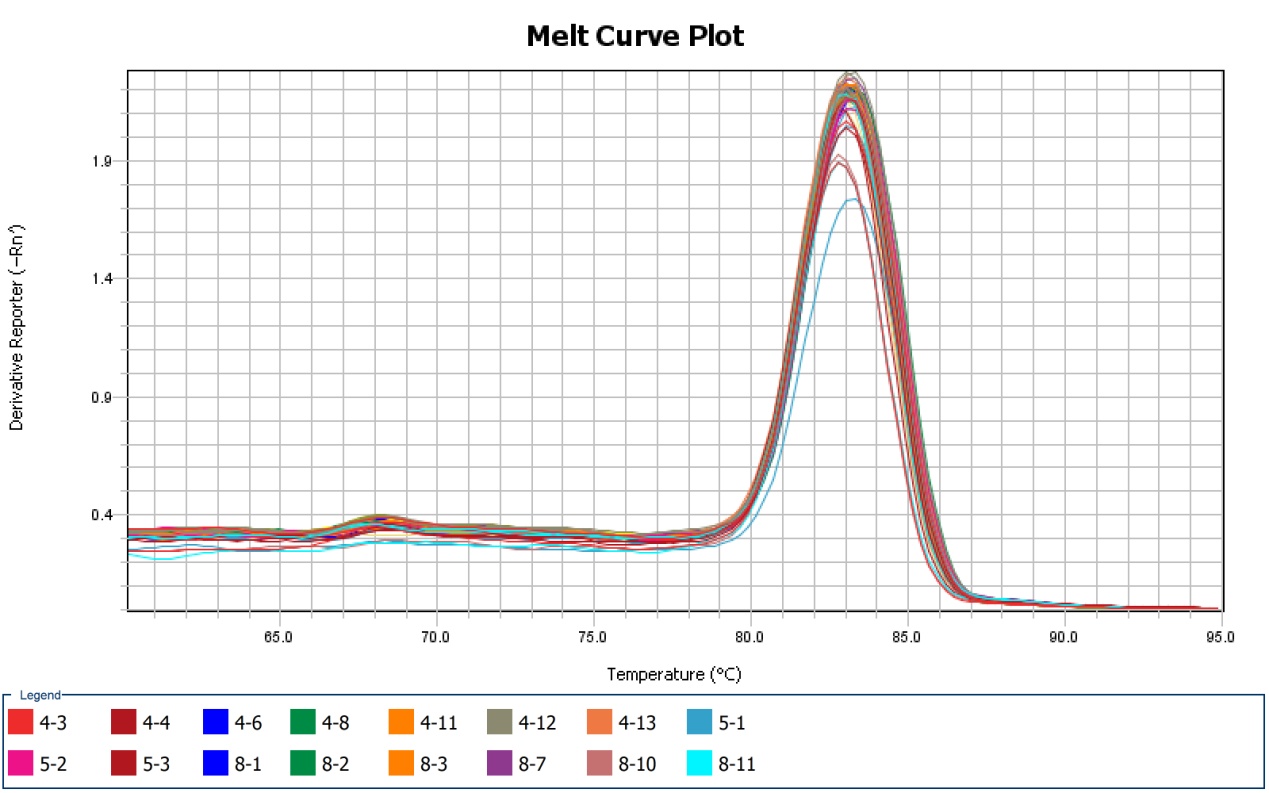
**

***Hsp70***

**
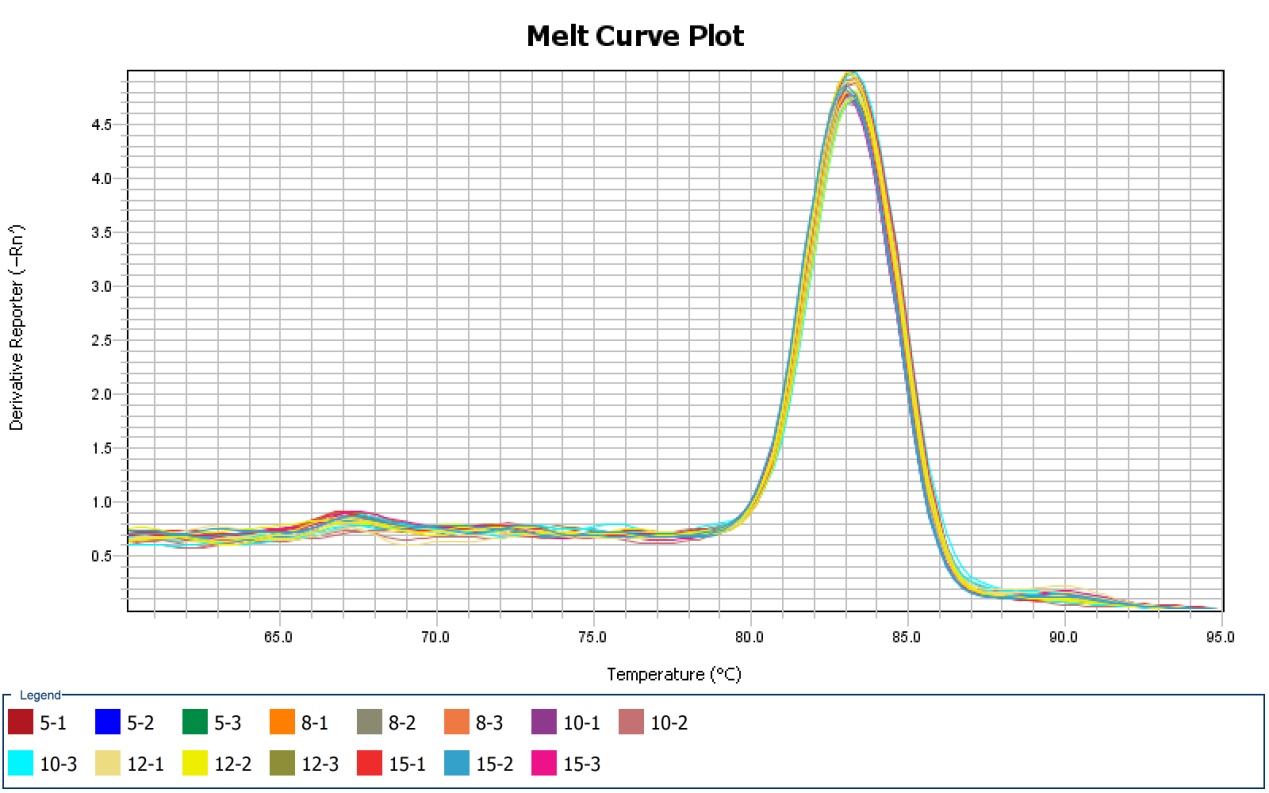
**

***PP2A***


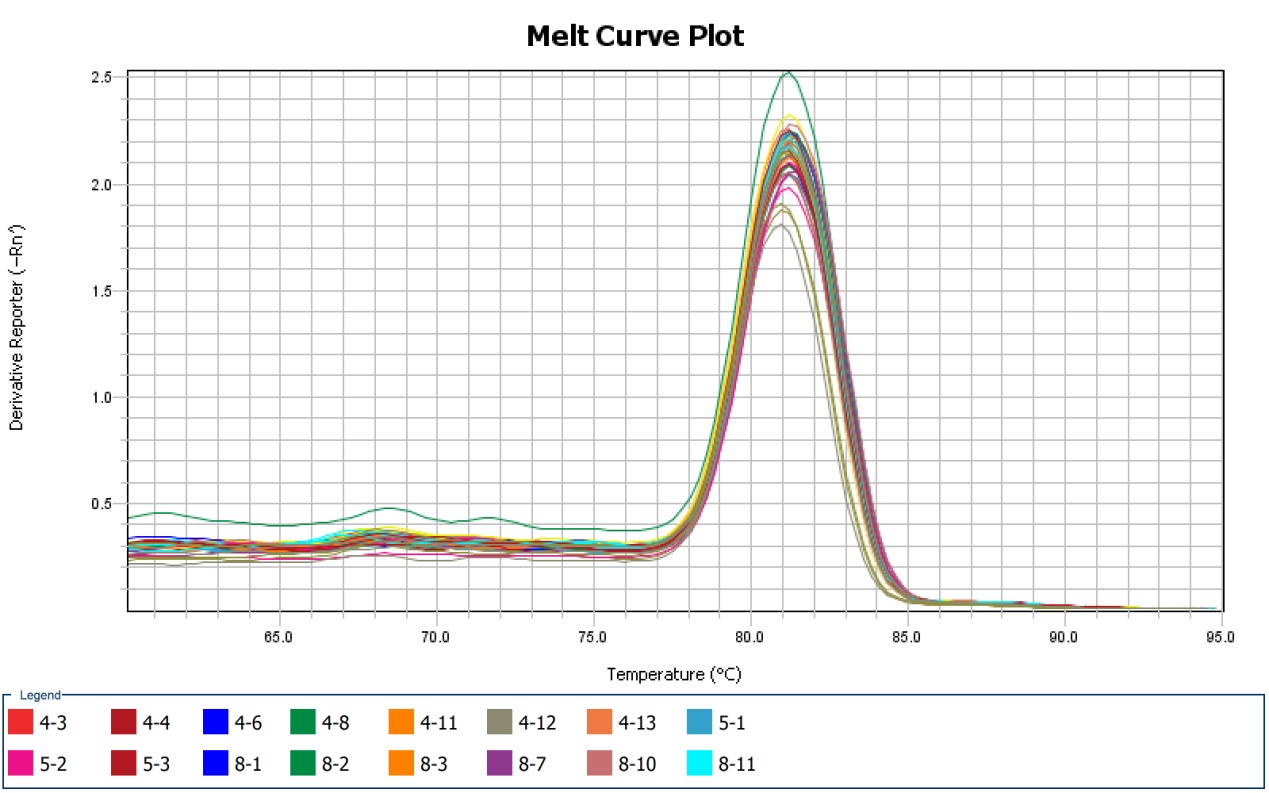

Supplement: Supplementary file 1 — Fig. S1. Melting curves for 11 candidate reference genes in 15 fruit samples. [file FEB4-11-3142-s001.doc]
