# Supplementary material for: Selection and validation of reference genes for qRT‐PCR analysis during fruit ripening of red pitaya (Hylocereus polyrhizus)
Source: FEBS Open Bio. 2021 Oct 6;11(11):3142–52. doi: 10.1002/2211-5463.13053 (PMC8564333; doi:10.1002/2211-5463.13053)
Supplement: Supplementary file 2 — Data S1. The sequences of 11 candidate reference genes and 2 target genes (SUSY and DOD) from RNA‐seq for red pitaya fruits. [file FEB4-11-3142-s002.doc]

**Supplementary dataset**

The sequences of 11 candidate reference genes and two target genes (*SUSY* and *DOD*) from RNA-seq for red pitaya fruits

***ACT7***

TCGCATTAAAGTCTGCGGTTTGGCAAGCCCATTTCCAATATATTGGCTTAACCATTGTATATTTGTATGTGTCTCATAAAGTGGAACCTCGAGTGTCAATCTTCCACGCACACACACATATATAAATGGGCATTTGTCGTTTTTCTTATTGCATTAAACAAATGCTTCAATTTATGTGTCTACCTCATCAATCAAAAGCCTATTCTCCAAAACTAGGCTCATTGAGAATCGCGTTGGACAAAACCCTCAATGTAAAAACTCCACAACTCCAACCTTTCCAAGGCTCATTCAATATTTCAAACGCACAAGACAAGCCTGGACCAATGGAACCGGCATTACCGGCCGAAAGACGTTAACTACATATCTTGTCAGAATTATCGGCCTGGAGTGTTAACAACCAGCTTGATTGTGGCATTGCTGAAACCACAAAGCAGATCTGCAGATGCCCACAAGGCTAACGAAACTCCCTATCCTGATATTCTCAATGCTTGCACACAAAGGCCCAGCCGGTCTCTATCAAATGTCTCATCCACAAAGGCGAACATGGTTCAGATGATCGGAATCATTAGAATTCGACAAACTACCAATCATAAGAGTCGAGTTTTTATGGCCACCCAAAAAACCAACCGAATCAGAATCACAATGGGTTTTCTCATAAGACTGCAACTATGGAACATAATGTGACAAAACACTTGCCTGACAAAAATCAAATTGAGGCTCAAGCAATGTGACCCAGAAAAATAGTAGCGTATGGATAAAAACATAGGGCAAATGTTCTGCAAAGATTACATGCCATGACCCTTGAAAAATAGTTACAACAAACAATAGAAAATCCAAGCCTTTTGGTTCCCAAAAACATAGAGAAAAAACAGACTCCTAAGTTTCCCATCTCATCTCAACCAAATCAAATAAAATCATACAGCACACTAATAGTTTGACCAAACGGAAAGAAAAATAAGGAGACAGCGGGGTACTATGTAGGACTAGGAACTCAAAAACTTATCTTAGAAGCACTTCCGGTGAACAATTGATGGGCCAGACTCATCATATTCACCCTTGGAGATCCACATCTGTTGGAAGGTGCTGAGTGATGCCAGGATAGACCCTCCTATCCAGACACTGTACTTCCTCTCAGGAGGAGCAACGACCTTGATCTTCATGCTGCTGGGAGCAAGGGCGGTAATTTCTTTGCTCATACGGTCAGCAATACCCGGGAACATTGTTGTACCACCACTGAGCACAATGTTACCATAGAGGTCCTTTCTGATATCAACATCACACTTCATGATGGAGTTGTACGTTGTCTCATGGATACCTGCAGCTTCCATACCAATCAATGAAGGCTGGAAGAGGACCTCTGGGCAACGGAACCTCTCAGCTCCAATAGTGATCACCTGCCCATCGGGCAACTCATAGTTCTTTTCTACTGCAGAGCTGCTCTTGGCAGTCTCAGATTCTTGCTCGAAATCAAGAGCAACGTAGGCAAGCTTCTCCTTAATATCACGGACAATTTCCCGTTCGGCTGATGTAGTGAACATGTAACCTCTCTCAGTGAGGATCTTCATGAGATAGTCGGTGAGATCACGACCAGCAAGATCCAAACGGAGGATAGCATGGGGAAGGGCATATCCCTCATAGATAGGAACAGTGTGACTCACACCATCACCAGAATCAAGCACAATACCTGTCAAGAACAAAAATGAAGCAATAATCAGACAAACACGAACAGAGAAACGTAATGGATGTAACCATAAAACAAAATGACAGGACAAGCTACAAACCTGTTGTACGACCACTAGCATAGAGAGAAAGAACGGCCTGGATGGCAACGTACATGGCGGGCACATTGAATGTCTCAAACATGATCTGGGTCATCTTCTCTCTGTTAGCCTTTGGGTTTAAGGGCGCCTCTGTAAGCAACACTGGGTGCTCCTCTGGAGCAACACGAAGTTCATTGTAGAAGGTGTGGTGCCAGATCTTCTCCATGTCATCCCAGTTGCTTACAATACCGTGCTCAATTGGGTACTTTAGGGTCAAAATACCTCTTTTAGACTGAGCTTCATCACCAACATATGCATCCTTCTGGCCCATGCCAACCATGACACCTGTGTGTCTAGGACGACCAACAATACTTGGGAAGACGGCCCTTGGAGCATCATCGCCAGCAAACCCAGCCTTGACCATTCCAGTTCCATT

***CYP***

AAGAGGAGGAGCAAACCAAGTTTCCCAAATTTCTTCAAAAAAATCCCTAGAAATTCTCAATCCCCAACTAAACTATGTCAAACCCTAAGGTGTTCTTCGACATGACCGTCGGCGGCCAACCCGCTGGCCGAATCGTGATGGAGCTCTTCGCCGACGTCGTTCCGAAGACCGCCGAGAACTTCCGGGCACTCTGCACCGGCGAGAAGGGAATTGGAAAGTCCGGCAAGCCCCTCCACTACAAGGGCTCGACCTTCCACCGAGTCATCCCCGGCTTCATGTGCCAGGGCGGCGACTTCACCGCCGGAAACGGCACCGGCGGAGAAGCTATCTACGGCGCCAAATTCGCCGATGAGAACTTCGTGAAGAAGCACACCGGTCCAGGAATCCTCTCCATGGCCAACGCGGGCCCTAACACCAACGGATCGCAGTTTTTCATCTGCACCGCCAAGACCGAGTGGCTCGACGGAAAGCACTGCGTGTTCGGTCAGGTTGTTGAGGGGATGGATGTGGTGAAGGCCATTGAGAAGGTCGGTTCTAGCGGTGGAAGGACCTCTAAGCCTGTTGTCGTCGCTGACTGTGGCCAGCTCTCTTGAGAATCTGGTCCGATCGGATATGATCTGATGCGGAATTTTTTGTTCCGATCATCATCATCATCCATCATCCAATCTTCTTTTCATCTTCAAGTAATTGTTAGGCCTGGGTGAGTAGAGAGTGAGTGAGGGGAGAGATTAATGTGATTGTCTCTGTCATCGTGAGTGTGAGTGAGTGAGAGTCATCTTTCGGGTGTGCGCGTTTGTGTTGTGGTGGTTCTCTCGTGGGCCATTCGAATAATATTAGTTTAATTTTCTCATCGATTTTGTCACACTTAACGTTGTTTCCGTCGCTTTCTTTGCGTATATATTTTATATTATCTGCGTTGTTAATTTCAGCAAGTTGCGATTAATTTTCAACCTTTGGAATTTTCAATGAAGATCAAAATATGGATTGAAATTTGGTATTTGCGGTAGATCTGGTGTGATCCAGAGCGAAAGTGAGGGAAGGGAAACAAAAAATAAAAATAAAAATAGTTTCTGCAAGAGGGAGGGTAGAGAAATGTGGAGAATTGGAAGTATTGACTTTTCAAAATCAGTGAAGGTTCTGACAAGTAAGCAGGGGCTCTGCTCTGCTCTGCTCCCATCTGCACCGGACACTGTAGGAGATTTTTATGGAGTCACGTGAGTCACGTCATGTTTTCGTTCAGCGTATGATAAGGCGGTTTTGTTTGGATCTTTTGGGCCTTGGGTTTGGGCCTTAAATGTTCTTCCGTCTGGTTGGGGATGTATATTTTCTCTATTTTTTCATCTTTTATTTTTTTTTTTGGTATGAGAAAGGTGTATGCTCATCACTCTATCTGTTTTTTTGGTCGATCATCTTGGTAGGATTATTTGTTAGTTTGTGTTTTTTTTTTGT

***EF-1α***

TCCTTCTTCTCAACACTCTTGATCACACCCACAGCCACGGTCTGCCTCATATCCCTAACGGCAAAACGACCGAGAGGTGGGTACTCAGAGAAGGTCTCCACCACCATGGGCTTGGTGGCAATCATCTTCACAAAACCAGCATCACCATTCTTCAGGAACTTGGGCTCCTTCTCAAGCTCCTTACCAGAACGCCTGTCAATCTTGGTCAAGAGCTCGGCAAACTTGACCGCAATGTGGGAGGTGTGGCAATCAAGGACTGGGGCATAGCCAGCTCCAATCTGACCTGGGTGGTTCATGATGATGACCTGGGAGGTGAAGCTGGCTGCTTCCTTGGCTGGGTCGTTCTTTGAGTCAGAGGCAACAAAACCACGCTTGAGATCCTTGACAGCAACATTCTTAACATTGAACCCAACATTGTCCCCAGGGAGAGCCTCAGGGAGGGCCTCGTGGTGCATCTCAACAGACTTAACCTCAGTTGTCAGACCAGTGGGACCAAAGGTGACAACCATGCCTGGCTTCAAGACACCGGTCTCAACACGACCCACAGGGACGGTTCCAATGCCTCCAATCTTGTAGACATCCTGGAGTGGGAGACGGAG

***EF2***

GTGCACCCCGGACTGTCATGAGGAAAAATACAACGAAAAGAACACAAAGGTTGTAAATACATATTTGAAATCAGAATCAGACATCAGTTAAAACATTAAGTAGACACCAGAGCCAAAATGACAAACTAGGGGGAAAAACCATGTTAACTGTCAGTACTGAAACACACAAGGAAAACCCAATCACAGAACATAGGATGCTCAGCAAGTAATAATACTAAACGAAACAAAATTCTTACTTCGAAAATTGGGATACTATCCCAAGCATATCAAACTAGTGCACAGTCACAATGTAACCAATACAGAATGCATATAGGAAACAACAATTGTTTTACTCAGAATAACTACCAAAATGAATTTTGTTCCATAGAGACGGAAAAGCTTACAGCTTGTCCTCATACTCGGACAGTGGGGTCATCTGCTCCTTCAAACCCTTCCTCTTACGGATATCTGCCACAATAGCAGCAGCTTGGGTTCCGGGCTCCAAGGGATCTGACGACAGCATGTCCCAGTGATCAAACACACACTGTGGGAAAGCTTGACCAGATGTTGCAGCCCTCAATGTACTCGAGAAACCAAAGGACTCAATGACGGGGAGGTATGCCTTGATGTTGTAGAGAGGAGTACCAGGCCTCTGCATTTCTTCAAACACATGACCACGCTTCTGGTTAAGAACACTGTAGATACCACCAAGAGCTTGCTCGGGAGCCTGGATTTCCACGAGGTAGACGGGCTCCATCAACCTGGGTTTGGCAGTCAGCTGGGAAGCGTAGAACACCCTCCTAGCAGTGGGAATAATCTGACCACCACCACGATGGATGGCATCAGCATGAAGAACCACATCGCACACCTCAAAACAGATGCCTCTCATGTTCTCTTCAGACAAGGCACCTTCTTTTGATGCCCATTGGAAACCGGCAACAACAGAATCCTTGATTTCATTCAGGTACTGGACTCCCTTACACATATCAACAACCATGTTGGGACCTGTAGTTTCTGGCCCAAAACACCAGATTTTCTTAGCAAGATCCTTGTCCCACCCAAACTCCTCAGCTAAGATCTTCGATCTGTTCTTGGGGTCATCTCTTGGACCAATACGGCCGTCATCAATAGCCTCGGCAAGCCCTTCCTCCATCGGACGTGCCTCCATGTACAAACGGTTGTGCTTGTTGGGAGACTTGCTCATCACAGTACGGCATGACCTCTCAAGAACAGTCTCACGGAAGGAGACAACAGGGTCAGACTTGATGATCTCAGCCCCACCCATAAAGTCCTCCTGCAGATCCTTCAAACAGATTTCCAAGTGAAGTTCTCCAGCACCAGCAATGATGTGCTCACCAGACTCCTCAATGGAACAGACAACCATAGGGTCTGATTTGGCTAGACGCTTCAAACCTTCAACGAGCTTGGGAAGGTCAGAGGCAACCTTACACTGCACAGCAACACGGACTACGGGGGAGACAGAAAACTTCATAGCACGGATTGGGTGGGCATCAACTTCCTTCTCGTTCGTAAGGGTTGCATTTTTGGTGATGTACTGATCCAAACCAACCATTGCAACAGTGTTTCCGCATGGCACATCCTCCACTGTCTCTTGCTTTTTACCCATCCAAATGACAGTTCTCTGAACGTTCTTGACATAGAGATCCTTCTTCTCTCCAGGCACGTAGTTAGGACCCATGATTCTAACCTTCATACCGGTAGAAACCTTTCCGGAGAAAACACGACCAAAGGCAAAGAATCTACCCTTGTCGGATGCAGGAATCATCTTTGAGACATAAAGCATCAAAGGTCCCTCCGGATCACAGTTCCTAATGGCCGTGGCATAAGGGTCATCCATAGGTCCTTCATACAAGTTCTCAACACGGTATTTCTGTGCCTTCGCCGGAGATGGCAAGTGATAGACCATCATTTCCAGCAATGCACTAGCAGCTGGCAGCCAGGTCTGCATGACACGCTTCATCAATGCCTTCCCCATCAAGTCTTTCTCTTCAGCCTTCAATTGGACACCGAGTTTTTGCAACATAGGCCACAGCTTGTCCTTTTGGTCATTCATGCAGGTTGAGATGATCTGCTTGATGGGCTCATAACAGAACTGGACAAATCCACGCTTACAAGTTGGGGAACCGGTGTTCTTGGTGGTCCACTTCTTAGTGGCTGGGTCAAAGAAGTTCTCACCCCAAAGGCGCTCCATCATCTTAGCCTCATCAACTCCAAACTTGGAGGCATACATCTTAGCGAAGTTGGACAGAGTAAATGCCCAACCATGGAGACCCGCTGAGAAAGCAACGGTTCCTTTCTCAGGATAGACTTGAACATCTCCCAGAAGGGGATCTTCATATGTAGCCATAATGACATTAGCATTCTCAATTACCTTCTGGAATGTCGTGTAAGCCTCCTCACCATCAACCTGAAGCTCAAGGAAGCAACGGTCCATCTTGTTAACAGTCAGGACAGGCCTGATCCTCTCACCAAGAGCCTGCCGAAGGACAGTTTCTGTCTGGACACAAACTCCCTCAATACAGTCAACCACGACAAGGGCACCATCAGTGATACGCAGAGCAGCTGTGACCTCTGACGAGAAGTCAACGTGCCCAGGGGAATCAATAAGGTTGATGAGGTACTCATTGCCCAGTCTCTCTCCCTTGTAAGCCTTCAAAGCTTCATCAGCCATCTCATAGTAGAGAGAAATTCCAGTGGACTTGATGGTAATACCACGCTCAGCTTCATCAGCACGGGTGTCTGTCATTCGCACATCACCTGCTACTTCCTGGGCGATGATACCAGCAGCAGCTACCAATGAGTCGGTCAGTGTAGACTTTCCATGATCGACATGGGCAATAACAGACATGTTACGGATGTTGTGTTTAAGGTCCATAATCTTGCGGAGCTCATCAGCTGTGAACTTCACCATCTTGGCAGATCTTTAAAAGAACACTGGCACTCAGCCAAATAGAAGAAGCTCCTAGGAGCTATTCAACCAACCCTGTGTAAAAATTTAACGAGTCATTGTACAGCATTGCATAGACCACCATACTTTAATACAAATGAAATATAGATATTTAAACAATACCCTTGAGGAAAAAAACGGAGCACAACTTTGAGCTCAACAGGCAAAAAGGAAGCTGTAATTCAACACATGTTTCAATTCTCAGGCAATGAGATGATTTTTTTAAAAAAAACATACAAGCAACAAGCAGTATAACCACATCCGACCATAACAGATCTAGTAATTCACCAAACCACGTAATAAACAATTGCACTAAATGAATATACCATACAAGCACTCCCGAGGCACCATGAATTAGTTGATCTTATCGGTAGTTGATCTTATCCGTACCACTACTGCCAAGAAAACTTTTGATCCAACTTATTTAGATGAACAGATATTAGCATTAAAACTGCAAAGTATGAACTGGGTCTATTAGGGTATTGTGATTCTCTTAAAATCTGCATCACCAAATAAAATAAGAGCACCAAAGTTCATTTAAACTGAAATGCCACTCAATGCTTCATATCCAAGCAAAACTACCAAAAACCTGCATTTTATTACGCACAACATCCGTTGAAATCGTAATTAAAAAAACAAATCCAACAATAAAGATAAAATTCAGCTCCTCTTTTACACCAGTCCTCAATTCTTCAGTAGATTCCCCGGATACGATACAGAAATTATATGAAGACCCTAAAAACAGACTAAACACTTCCAAGGACATCAAATTGGATCAAATCGAATCAAATTAAATCAGATTAAAAAGAGAACAATTTCCTCTTCCCACGGGAGCCAAATAAAATTTATAACAAGTCAATCATCAACGACTCAACATCAGCGAAGACGATAATCGAAGATAGAGGTACCTGAGAGAAAGCTCGAAGCCGCCGGCGAGAAGGCGGTGAGCTGGTTCGTTGTCCGATCCAAATGTAGCGGCAGAACCGCAGAACGTGTGCGGGGAAATGTAGAATAAGACTTCAGAGGCAGAGACAGCTTATATTGGCTGC

***IF-4α***

CTTCTAGTATTAACAAAGATGACACTCTGGGTGATAGCCAATGTCTCATAAAGGTCACAAAGAGTGTCGAGCTTCCACTCCTCCTTGTCAACATTAACATAGAACTGCTTGATACCCTCAAGAGTAAGCTCATCACGCTTCACAAGAATCCTAACAGGTTTGTTCATGAACTTCCTGGTGATCTCAAGGGCCTCAGGGGGCATTGTAGCAGAAAATACTCCAACCTGGATCTTGGGTGGAAGCTGCTGGAAAATGTCATAGATCTGGTCCTTAAACCCTCGAGAAAGCATCTCATCAGCCTCATCAAGGACAAACATTTTGATATGGTTAGCACGAAGAGATTGTCTCCGCAACATGTCAAAGACACGGCCAGGAGTACCAACGACCACATGTACTCCAGCAGAAAGGATTCTTTGATCCTCACGAACACTGGTTCCACCAACGCAGGCATGAACCTTAACGCCCAGGTAATCACCAAGTGCCCGCATAACCTTCTCGATCTGTTGAGCAAGCTCTCTGGTAGGAGCGAGAACAAGAGCTTGGCATTCCAACAAATCATAATCCAGCTGCTGTAGGATTCCGGAGCAGAAAGTGGCAGTCTTGCCGGTACCTGACTGGGCTTGCTGAATTACATCAAGACCCTTACAGAAGGGTACAATTCCCCTTTGCTGAATGGCAGAAGGCTTCTCAAAACCGTAAGCATAGATTCCCCTAAGAAGGTTCTCTTTCAACCCCATCTTATCGAAGCTATCATAGACCTCATCATATGAAGTGAAGAAATCTTTTCCTTCCTCAGAAAGCAGTTCATTCATTTTGGTGTCAAACTGACGAGCGTCAAACTGAGATCCCTCGGGTGCAACTCCAGCCATGATTGATGAATTTGGATTGAGAAGATAGCAAACAGCCGCAGCGTCGAAAATTAGCGCTAGGGTTAGAGCCTTACGAGGAACTCTAACCGGATCTG

***GAPDH***

ATAAGCCTAAATAGATTCATTACTAGAGAGCTACCGGGCTGATTAAACGTGAAATTTAAACTTAATCCAAACTGAATTTTCACCTCAAGTTGATTTTCTTTTATTAGATAAATTTAGAGGACAAACTTCAACCAAACAAAGATCAACTTAAGCAGAAAATAACTCAACTCAAATCCCAAACAAAAGATCGTGTTTTCAGGGATGCAATGAGAGCCAGTTCTGAGTCTCTTCTTTTTCGAGGGGACACGACAATAAAAAGTCTGTCACTTGACATAGACCTGGAGGTGATATCCCCTTTGAACATCATCTCTGACTTGTCGGTTGTATGTCCTCGTTCAAATTTCCTCCGAAAATTTTCGAGATACCATCACAACTACAACTGATGAAGAGCTTTCAAAAAAAATATTGCCAAAATTGTCCCCTCCAAGCAAAACAAAAAAGAATATTTGCAGCCCGCTACGAAGCACACGGACAACCGCAAGGGTGGCAAGCATATAGTATAAACACTCGTCATATGACAGCCGAGAAATGCCATGTTACAAGAGAAGGAAAAAAAAAACAACGCAGAGCGGCAGCCCATAATGGCTGGAGCAGATTGTAGTTGCAGCTCTCACTGGTCATTATTATCAATATTTGGTGCCTCAACAACCACAAGGGTGGCAAGCATCACGAACAATAAAATATCATGCAGCTAATATTGCATCAAACTCCAACCTACTATCTCATCCAAAACCTGTTTCCCCAAAAAATTGTGAAAATGGGTAAAACTCGCAATTTGTGGTTTGGAAGAATTAGCTAGATGCCACTAACGCCATGTGCTCAATCAAGTCCAAAACTCGGTTGCTGTATCCCCATTCATTGTCATACCACGAAATCAGCTTCACGAATGAGGGGCTTAGGGCAATACCAGCCTTGGCATCGAATATACTAGATCTGGAGTCACCAAGGAAATCACTCGAGACAACATCATCCTCTGTGTATCCAAGAATGCCCTTCAATAGCCCCTCTGAAGCAGACTTGATGGCTGCTTTAACATCTTCATAGGTGGCATTTTTTTGAAGCCGACAAGTCAAATCTACAACAGAGACATTTGGTGTTGGTACACGAAATGCCATTCCGGTAAGTTTTCCATTGAGGTCCGGCAAAACTTTTCCGACGGCCTTAGCTGCACCAGTAGAACTTGGTATAATGTTCTGACCGGCACCACGGCCTCCTCTCCAGTCCTTCCTTGACGGTCCATCCACAGTTTTCTGGGTAGCTGTCGTAGCATGCACAGTTGTCATGAGACCTTCAACAATCCCAAACTCCTCATGGACCACCTTTGCCAGGGGAGCCAGACAGTTCGTGGTGCAGCTTGCATTGGAGACAACATCCATTTCTGGCTTATATGTTTTCTCGTTTACACCTACTACAAACATAGGTGCATCAGCTGATGGAGCAGATATTACCACCTTTTTAGCCCCTCCCTTCTTATGCGCAGATGCCTCCTCCAATGCTGTGAACACCCCAGAAGATTCAACAACATAGTCAACACCGTAATCACCCCAAGGGATTTCCTCTGGGTTCCTGTAAGGCAATACTGTAGCTATGCAGCAGGATACAGAACAAGATGGCAGCACTCCAACTCTATAAATTTAAATAATTGCAACCTGTCTAGAACAACACTAGGATCAATGCCAGGAAACTACCTTTTGTTTGTAACTTTGACGTGTTTCCCATTTATGTCCAGAGTCGAATCATCTATAACCCTTATGTTCCCATTGAAGACTCCGTGGGTTGAATCATATGTAAACATATAAGCCATGTATTTGACATCAATGAAAGGATCGTTCACTGCCACCACATCCATATCATCCCGTGAAGTTGCTATCCGATGCACCAGCCTTCCAATGCGACCAAAACCATTGATTCCCACCCGGATCTTCCCACCGCTCCTTGATTTTGGAACTGCTGATGGAATCTCGGTGGCAGTAGCTTTGATAGGCTGCAAGCCTGTGGATCTTGATTTCCTGCAGCAATGGGAGGAGGAGTAGCCGCCATGAATAGCAGTGCCGAAAATGCTAGTCTCACTCTTCAATGAACTAACATTCCGATTGAATCCACCGTACGAGACCTTGTAAACATCGTAAGGACGACGAACAGAGAACTCAGAACACGAGTTCTCGAAGAGGAAAGTCGAAGGTGCAGATCTTATCAGAGAAGAATACGCCATTTTGCTTCTCTCCGTAGATCAACAACAGCACAGCAGCAGTGGATGAAGAGAGAGAAAAACCCTAGACAGAGGAAGAAGAGGGAGGGGGCGAAGGTAATAAGGAGATGCGTCTTCCTCTGTCTTTCTGTCTGTTTGATGAAAATGTTGAAAATGGGGATTGGGGGTTGAGAGGGCAGAAGTTGGTATTTTTTATAAAAAATACAAAGTAGAAAAAGGGGCACTCTGATTTATGAGGAGAGAGAAAGTGTGCTTTCGGACTGCTGCTGAGGCCTTGTGTATCATGAAGAAAGCGACGGGTACTAAAAGAGTACTGTTTGAAAAACCAGCGACGGGTACTGATCCGTCGGAATTGTTTAATGAATTGTTTTCGAAAAACAAGCAACGGATTCGTGCGTTGAAAGGTTTTCACTTGAAAAAGC

***TUB***

GCCTTCCTCATACTCTTCCTCATACTCTGCAGTTGCATCCTGGTATTGCTGGTACTCAGCCACCAGATCATTCATGTTGCTCTCTGCCTCTGTGAACTCCATCTCATCCATACCTTCCCCAGTGTACCAGTGCAAGAAAGCCTTGCGCCTAAACATGGCTGTAAATTGCTCACTCACCCTCCTGAACATCTCCTGAATGGATGTGGAGTTGCCCACAAAGGTTGAAGCCATCTTTAGGCCAGTTGGTGGAATATCACAAACACTTGACTTGACGTTGTTGGGGATCCACTCTACAAAGTAGGACGAGTTCTTGTTCTGAACGTTGATCATCTGCTCGTCCACCTCCTTCGTGCTCATCTTTCCTCTGAACATGGCTGAGGCTGTTAGGTACCGACCATGGCGGGGGTCAGCAGCACACATCATGTTCTTGGCATCCCACATCTGTTGGGTCAACTCAGGCACAGTCAAGGCCCGGTATTGTTGGGAACCCCTCGATGTCAAGGGAGCAAACCCAACCATGAAGAAGTGGAGACGAGGGAATGGAATAAGGTTCACGGCTAGTTTCCTGAGATCAGAGTTCAGCTGACCAGGGAACCGC

***DNAJ***

TATTTGGCCTCTTCGTTGCGGGAGAGGGAGACTAGTATGCCTGCCATCTCCCACTCTTCCCTCACACATTCCAGTTTGATTCCAATTCCGCATCCAAGGCCTGGTCTTCTCAGGTGAGTCAATTCTCTTCTTGATTTTGTATTTTTGATTTGATTTCATTTGATTTGAAAGCTCAAATCCTTATTTTCTTATCAATTCATGGTGCTTCCATTCCAATTTGGGAATAATTCCTTTTCGTGATGCATTTCTTACGGAATTCAACAAACCCCTAATTTCGATTCTGTTCTTCCGTTCAACTCTTAGGCCGAATTGATCAGTATTTTAGATCTGCATACTGATCCTTGATCGCGGCTGTGGAATTTGGTTTCTTCCGGAATTTGCGGTTTTTTTCCATTTGAGTTGATAGCTGATTTTGATTTCGGCTTGTGTTGATTGGGTTTGTGTTTCCTGAATTGAGTAATTAGGGCTTCGATTAGGTAAAGCCCTTTGTGAATTAGGGAGTTATTATTAGTTGGTTGGAAGTCATGGATGGTAACAAGGATGAGGCGTTGAAGTGCATCGGTATTGCTAAAGAAGCGATTGCATCTGGGAATAAAGAGCGTGCGCTCAAATTCATCAGAATTGCGCAACGGCTCAATCATAATCTATCCATTGATGATCTGCTTGCTGCTTGTGAAAAAATCGATAGTGCATCCCCGAGCCCTTCTGAGAATGGGGGCCATGTTGAGAGAACCAAGAGTGGACCTGTTAAAACTGAGGAGACTTTGAATGGAGAGCGGAGTTATACTGAAGAACATGTTCAGTTGATTAGGCAGATTAACAAGCAAAAGGAGTATTATGCTATTCTTGGGGTAGAGAAGACGTGCTCGGTCGAGGAGATTAGGAAGGCGTATAGGAAACTGTCTCTCAAGGTTCATCCTGATAAGAACAAAGCTCCTGGCGCAGAGGAGGCATTTAAGAAAGTCTCCAAGGCTTTTAAGTGCTTGAGCGAGGAGGATTCTAGGAGGCAGTATGACCAAACTGGTTTGGTGGATGAATTTGAGTATAACCAGCAGCATAATGTTCGAAGGCGGAGACGAAGGACAACCACTGATTTCTATGATGATGATTTCGATCCTGATGAAATCTTCAGGGCCTTTTTCGGTCAAGATATGTTTCGCACTGCTCATGTCTATAGGACAAGGGGAATGGGCACACAGCAGAGGACAGACTTTAATGGAAGAGGTTCTGCTTCTAATGTCATGATGCTTCTGCAGTTGGTGCCATTCTTGTTAATCTTGCTGCTTGCTTACTTGCCCTTCTCAGAGCCTGAGTATTCTCTGCAAAGAAACTATTCGTATCAAATCCCAAGGGTGACCGAGAAATATGGTGTTGATTACTACGTTAAATCACTGGACTTTGATCGCAGTTATCCAGTAGGAAGTGCTGCACGAGAACATATTGAGGAAAATATAATTCAGGAATATAAGAACTTGCTTGGACGATCCTGTCACATTGAACTCCAGCGGCGTCAGTGGAATCGTAATCGTCTTATGGCAACCCCATC

***PTBP***

CAGGTCCAAATGCAGAAAAGACCGTGTGCAAGACATCCAATGTCACAGCATACTGCATGTTTTCAATTGATGCTAGGAGAACATTACTCTCAGGTTCTAATTTTTTACCATCCAAGCCCACACCTAACTGACCAGTAGCATCTATGGCTGATGGGGCAACTGGGAGGTATGGATTAGTATAATCCCTGCTGCGGTGGCTTTGGAATTTGACGCTTAGATCAGTATGTGCAGAGTAAGTTATTTTCAGGCTGCAGGGGCCTATATGCTCCGGAAGCAGGTATCTTGGAATGCTTCTTCCATCCAGAGCATTCTTTGCAGAAGAAGCAGTTTCTGTGTCAGAAAATTGAATCAAAGCCTGAAACCCAGCTGTCTTCTCAAAAGTTGTAATTTTATGAACAAAACCAAATGCTGAAAATACATGTGCTCTTTCTCATGATGCAGTAAGCTGCCAACGAAGACATATCCTTGACAATGGCCTGCAAGTGCAAAACATCAATGCTGACTTGACGAGCTTCATTTCCCTCAATCGTTACCAGTAGCACATTTCCAGCAGTATCAGCAGTAGTCTTATTGTTTACAATCTCTTGCCTGTTTGAATACTGCAAGTAAACAGTTTTCCCCCGTATTTGAGCAGGTTCTGAGGAGGAAGCATAGTAGGATATCATTGCAATGGCTTGATTCAGGTCCGCCTAGAGCAAAATGCACAACACTAAGCACAACATCAAGAAAGTAAAGCCAATAATTAAATGGAAGACAAGAATACAATCAATAAAATGAAAAGGATTTCTTTGATCCCAGACTTCTTGCCCGAGCTCCCATCTAGTGTCTATCTAACAGGAAAAGACCCTAATAATAGTAGAC

***Hsp70***

TAATCTGCGCCCAATATGGTCACATTACATGCCATGCACCAGCACATGGCTTACGAGGCACAATTAAGAATCTTACTGTCCGATTTGGTAAGCTTAAGAAATCAACATCAAACATGCACGGCTAATGAACGTCAGACATTTAGCATTAGTTGGTCACAACCCTTAAAAGGCATTAAACATGCACATGGTTCGCCGGTGAAAACACAAACAAGGATTTGACTAGTACCCTTCTAAATCATTGGGCTTTCTGGGCATGGCCACCCGACGTTCTGCTTGAAGTTTCTCCGACCGCATAAATGCTCTCATTTGGTCGAGAATTTTTGCTAGAAGCCATCAAAGCACCACATAGATGCTATAGTACAAAATAATACCAAGTGTACTCTATTTGCTATTTTCTGAGAAAATCTGTCTATCCGACCTACATTCCAGGAAAAGAACTATAAAAACCGGGGAGGGGGGGGGGGTGTATAAAAAAGAGCGTGATTGAACGAGGTAAAACTGACTTGTCAACCCACACATCACATAAGCATATGCCATGGTGTGTTAACAAGGTGCACCACTGCCTAGCCTAGCCAGGGAACTCATCAACTGATGCCAAATGCCGGAAAACGTTGGCTCCATAATTAAGGGCCATTCTTTGAGAAGATCGCATGCCAACAATGGATGTCTTTGGTGTGCGAAGCTCCGCTATCATCTACCCTAAATAAGCCTGGTCGTACAATCACAATTCATCGTGAGTGGGAGCCTGAGATTCTGCATTGCCTGATCCTGATTCATCGGAATTTTCAGTTTCCTTGGAGGAATTATCCTCACTAGAGCTGGGAGTAGAACTGGAAGAGGCGTCCTCTGCTTTTTCGCCATTCGTGTTGTTCTTAACAGGCTTCGTTACTTTTGGTTTGGGTTTTGGGATTCTGTCCACGATGGCAACCTAGAAATATATATCAAATACTTATTCAGAAGCTTCACACACCAGAAAATTGTCATTAATTCTTCTGCAAAATAAGCATTTGCCTTAATGCCTATATTTTACCGTTGCTTGTCTATCAGCCAGCTTCTCATACACTTCTTCAGATGTGAAAACAGGCGTGCTTGATGCAGGAGTCTTTTCCTGCTCAGCCTGCTTATCATCCAACCACTTCCTCAATTTGTCAGCACTACTCAAAACCTCATCAATTCTGTCCTTCGGAATCCAGGGCTTCTTCGTCTGCCAGCCACGAACAATCTGCAGTTGTGGAAGAATACATGTTGGTAAATCTTATTATTCAGCTGCCTTAGCTGGGAAGGAATATTTGCACAACAAAGAACGTGAAAAACACATAGCCAGTTTACCCCCTTTCCCTCCTTTTTTTTCTTTTGCTTTTTTTTGGGGGAGGGGTGTTAGCTTTCACTCCAGGACCACTGCAAGCTGTCGGTGGCAAGTGGCAACTCATACCTCTTGTAATTCATCAAAATACCTTCTAGCCTGCTCAACAGCTGCTGGCCGAGCAGTGAGTTCATTCAACCTGAAAAATATGGGATCACCAATAGCTTTTAACATATTAAGACGCTCTTCAAACTCTCCGGCTGCAGCATCTTCCCCATCCATATACAACCAATCTTGGACCTCTTCAAGCTTTGCAATAAAGGACTGCCGCTCTTCACTAGTTGAAACTTTTTCAATCTCTTCTGATGATTCAATCTTTTCTCTAGTTGAATAGATGTATCCTTCTAGATTGTTTTTCAACTCCGCAGTCCTCTTTCTGTCTGCATCTTTCCTGTTCAGTTCCATCAGCTTGCGCTTAGAATCAGCCAGAGCTTCATCTGAAAGAGGCATGCCCAGTCCAACTGTCTTCTCAACAACCTTCAGTGGAACCCTGAAAGTTCGCTTCTTCAATTTCTTCTCTGTGACCTGTGCAATATTTTCTTCTTTTTCACTGGAGTTGGAGGGATTGCTTGTGCTACCATCAGAATGTAAGCTGCCATTCCCTTCTTCAGCAGAATCCCCAGAACCAGCTTCAGCTGATGAGTTAGCCGAAGCAGAAGCTTCAGCTGATAAGTTGGCAGAAGCTGTTGAATTCTCCAAAGTCAAATTCTTTTTAGGAACTTCTACCCACTCAGTTATCTCAATAACAGCATCAGCACGATCTAAAGTTAGTACTCCGCTTCTACTCAAAGAGAAGTGCAGATTTGCTTTGATTGGTGCAGAGAGATTCCGGGATGAATATTTCTCAGTGGCATTGGCCAAGCCAGATACTGCATATTCAGCAAACACCGGAGAAGTGACACCAGGTGGCAAGAAGTCTGCACTCTCATAGGCCAGAGATACTTCAAAGTCTTTGTCATGGATAATTGACCGAAACATCTTGCTAGGGACCTTCTTCATACGCTGTACAAGGACTTGCTTCGTGTTCTCATCTTCCACAAGATTGACACCATCCAACTCCATGACATAGCCATACATAGAACCATCAATCATTCCCAATTTTCTATTTAATTTTATCCCATCACTCAGATTGGCTGCATGTAATGCTGCACCAAGAACTATAGCCTCATCAGCATCAAGATGTTTATCCAGCTCTTTCCTTCCAAGAAACTCTTGAAGCTTGGCCTGTAACTTTGGCACTCTAGTTGCTCCTCCAATCAATTCTACTGCAAAAACATCATCAGTTTTCAACCCTGAATTTTGAAGAACTTCTTTCAGAGGCACAAGAGCTTTCTCCCATAAGTCCTCACACAGCTCCTCAAACTTCTCACGACTTATAGAGCTCCTGAAATCACGATCATCGTACATAGACTCCACAGATATTGGAGCCATTGTATTTGCACTCAATATTTCTTTAGTACGCTTGACTTGCTTCTTCAGTTTAGCCATTGCTTTTGGAGACTTTCTCACATCAACCCCACTTCCGAGTTGCTTATTGAACTCATCAGCAAAATGCTCCACCAACCTCATTTCCATATCTTGACCACCAAGCTCTGGGTTCCATCTGACATCCCTAACCTGAAACTGATTGATTGAAACAGTCTTTCCATATTCCTTGGTGTTATACGAGGAGAAATACACAAGAGCTGCATAAGTACTGCTCGAACCCATATCGTAAAACACAATATTCCTCGATTCATTGGCAAAATTCTTATCAATTCCATACTGCAACGCAGCTCCTGAGTGTTCATTAATCAACGAAAGCACGTTAATCCCTGCCAACTGGGCAGCATTAATCAAAGCCTTCCTCTCAGCTTGCCCAAAGTACGGCGGCACAGTGATTACCGCGTCTTTCACAGGAACCTTCGAATGAAACTCGGCCAAATCCAACGCATAGCTCAAGATCATGGCCAACATCTCCTCCATTGAATAAACAGCTCCATCATCAACCTTTATCGCTGCAACACCCCTAGAATCTTCCACGATTTGATAAGGCAAGTACAAGAAATCGAGCCTTTTCTTGGCGTAATCATAAGGTTTCGCAATTAGGTCGCGAATTTGGGCGTAAACTTTATCAGGATACCTAGCGATCAAACCAGCGGCTTCTTCGCCGATCAATCGATTACCTGATTGAAATGCCACTAAAGAAGGCGATTTACGCTTCGACATTTCGTTGATTGCGACCGAAATCGGAGGTTGTCCGGGCTTCAAGTTGACGACGGAGACTTTGAGCCATTCTGAACCTAGATCTACGCTGAGTACAGCCGATTGAGACTGAATTGCAAACAATGAGAGTAAGCATAGAAGGACTAACCCTAATCTGAGTAACATTGTTGATGATCAAGCCGGAAACGCAAAGAAATTTAGGGTTTTAACGGTGAATTGATGGATTCACCTTGATTTGATCCAGCGTGAGCGAGTGAGGCTCGGGATTTCTCCAGAGAGAGAGAGAGGAGAGAGGGAG

***PP2A***

CCTCAACCCCTCCACAGCTCCACCCGAGAGAGCGTGAGAGCGAGAGAAGCGCCTCTCCCCTTTCCTGAGAGTTGGAATGATCCCCAAAACTATTCAAACATTGATGAGATTTTAGAAGATGTGAGAATCTGAATTATAGCTAGCTGCTCCACCTCCTTTCTTTAAGCTTTTCAACCATGGTTTTCTGAAAGCTACTCCCCAACAAACCCCCCTCCAACCCCCGTCAAATATTTAGCCTTTTTGTCTCTCTGACCTCCATTGATGGATCTCCTTTAACTACTCAAGCTCTTAGTTTTGCTTTTCAGGATCTGGGTAACATATCTGTCTCTTTGTCTCTCGGTCTCTCACTCCTCTAGATTTTGCCTGCATTTAGAGTTTTGAAACTCATATTTTTCCTGTGTTTCTTTTCCTTTTTTCATTGGTCCTTTTTCTCTTTTTCTCCCCTTATCTTCATACCCAGTTGTCAATTTTTGAATTATCTCTTCCATCTTCTCATTTGTTCTTGCCTCTGGCTTGGGATTCAAGGGAAGAAAAAGGGCGAACTTGGGGAGGGTTTCGATTTCCAGAATTCAAATCTTCTGGGATCCAGATCTGACTTACAGCCATATTCTTCACCTTTTTGTTCATTCTTTAAGAACAATTTCCATTACCCACTTTTTGTTTCAGCTACTTAAGCTTTGTTCTTGCATGGTCTGGGATTAAGATCTGAGAATCAAGGCTACCTAGTGGTGTAGGATCTGCTTTTTTTTCTGGTAATTTATGGTATAGCATTGTGATTCTCTTTGATTCCATGTCTGTGATGGGGATCTATCATGTTAGAGGGTCTTTTGATGTTGCGGGTTTGAGTTGATTGGGTTGAATTCCACAAGAAATTTGTTTAAATTATTCACCATGTTGAAGCAATTCCTCAGTAAGATTCCAAGAAAGTCTTTCAAATCTGATAATGGGGACCTGATGTCCAACAACAATTCAAATAATTGTGGGAATGGCACTCCAAGGGCACAGAATGGGAATGCTGTCTCAGGCAAAGCGAATGCTGTGAAACGAACTTCTTCAGCTGTGTTCCCTACCAGTGTTGTTGCTGGAATTGAGCCCCTTGTCTCATTCAAGGACGTTCCAAATTCAGAAAAGATGAATTTGTTCCTGAGCAAGTTGAACCTGTGTTGTGTAACATTTAACTTCTATGACCCTAGTAAGAACACTTCAGAGAAGGATATCAAGCGACAGACACTCATTGAGCTCGTGGATTTTGTTGCTTCGGGTCCTCCGATGAAGTTCCCTGAGCCTGCTATCTTTGCCATGTGTAGAATGTGTGCTGTTAATCTATTTAGGGTCTTCCCACCAAATTATCGATCTGGTGGGGGTGGTGGTGAGAATGATGATGATGAACCGGTGTTTGATCCTGCATGGTCACACTTGCAAATTGTTTATGATTTGTTGCTTAAGTTTATTACCTCCTCATCGCTTGATGCAAAGGTAGCAAAGAAATACATAGACCACTCGTTCATTTTGAAGTTGCTTGATCTCTTTGATTCTGAAGATCCCAGGGAGAGAGAATGCTTGAAAACTATCCTTCATAGGGTTTATGGTAAGTTCATGGTCCATAGACCCTTTATTCGAAAAGCTATGTGTAATATTTTCTACCAGTTTGTGTTTGAGACTGAGAAACACAATGGAATTGCTGAACTGTTGGAAATATTCGGTAGTGTCATTAGTGGCTTCGCATTGCCCTTGAAAGAGGAGCACAAGATATTTTTGCGGAGAGCTTTGATTCCTTTGCACAAACCAAAATCTCTAGGTGTCTACTTTCAGCAACTGTCGTACTGTGTTACACAGTTCATAGATAAGGAGCCAAAACTAGCTAGCACTGTGATAAATGGTTTGTTGAAGTACTGGCCAATTACAAACAGCCAGAAGGAGGTGATGTTTTTGGGGGAGCTAGAAGAGATATTGGAAGCTACCAACATGTTTGAATTCCAAAAAGTCATGGTTCCATTGTTTTGGCGCATTGGACGTTGCATCAACAGCTATCACTTCCAGGTAATATATTCATTCAAATCTCTAATGCAAATTGTCCTGCTGCTGTGCGTGCACCTCCTTATATCCCATGCCAAGTATGCATACATGCGCTCTTTGATGAGTAATGAAAGTCACTGCAAATTCTGGTATCTCAAATGCCACTGGAGTTCAGGTGGAATTTTTTTTGGTCAAGGATCTTCAGGTGGAGTTTTGGCTCTGTCTGTCCTCCTTTTTGGGAGATGAGGCTGAGCTGACACAGCCTGTATCAGCATACAATCAACTGGCTGCAGGTCATTATGCTTAGGTATTTGAAATGGGTATACTTGAGGAGAAAAGGGAAGATCAGTATAACTACTGGTTGTATGGCATGCTTTTGACCTATGTTACCCCTTAGTTAGACTCAGCAAGAACGGGCCAACCTAGATACATGTCTAGGTGTTGGAATTGATAATTTTCTGAAAAGTTTGCACTTTTTTGGTTTTAATTTTTTAAAAGAAACCCTTCAAATTAAAAGGCCAAGTGTCCGTGTCCATGGTGGAGTTTCAAAGTTCCAGCATTGGTTCTTGGAAAGTCCGATTGGCATAGTTTTTAACTTGATTTCTGTAATATTGCATCAAAATCTGCTTCTAGGTCAGAATATACTTCAGAATTTTGGGTCCAGATGCTGGGGAGAAGCCGCATTTATGGGCCTTCATTGCATCTGAGCTGTGTTGAAGATTTGCAGCTCAACATCACATGGGTTAGGATTTTCTGGGTTACTTTTTGGAACAAAACAGTTCATTGCATATTGTTTGGATTAAATGCACATGTGCAGCGTGCTAGATCATTATCTGATAATTTTGTTGTAAAGATGCATAACCTGCTTTTGATTCAGCAGTTATTTTTGTGAAATGGTTGTTTGATTATCCACGTATAGAATTCACTTGCCAAGGATAGCTTATCTTTAAGTGTATCTGGGCATGATTTAACTAGCATGTAATCTCTTCTTTAGTTCATTGGGTCGTTCCAAGTTGGGCCTGTTTTGATGAGCTAACTTTGGCAGAGGTGTTGCATATGGATCGCAGGACAAACACCCGAATTGGTTTATTATATAAATTTGGCTCAAAAAGAATTGAAAGACCTGGTTTTTTTTTATCTGAATAGTTTCCCCGTCTTGAGATAGTATCTTGTTTCATCTCTCAGTAGGGGTGTGATAATTAGGGTCTTGAATTAACTTAAAAGGTTTCAGGGAAGGTTCAGATTGCTTTATCATCAGATGGGCACTCCATTGTTTCAGATGGACTTGATTCAAGTATTGCATATGTATCTAGCATGTTTGCCTGAGATGGCTTAATGTGCCTTCACTATAGCTAGTGAAATAGTTACTCAACCTGAACCACCTGGAATGAAATCCAATTCAGGACCCAAAATAACCTGTGACCCAAGCCTGAATAAACCCCGATTCAAACCAGACACCACCCAAGATTCCATTGGCTGGAAAAGGACTCGTCACAAAATTAACATGACAGCTATTCAAATTGAGTAATCCTATTTAGCCT

***SUSY***

CTAATTTCTTGCTTTGTTTAAAATTATTGATCAGAAATGGCTGCTAAATTGACAAGAGTTCCCAGCCTCAAGGAGCGTTTGGATGAGACTCTTACTGCTCAACGCAATGACATCCTCAACTTCATTTCCCGGTACTCTTTTTATTTTTTTTTCTTGTGAGAAGATTTTTCAGAATTTGTAATTCTTTTTTTGGTCAGTATCCTGAAATTTACAGCTTAACCTTGTCATAATCCAACAATTATGCAATTTTGTCTCTGTTTTTGCTTTTTCTGAAGTGGGGTTTTGGTGGGGTTTTAACTTTTAATCACTCAAACTGTCCAAAGTTGGAACTTCTTTCACTTTTGTCCTTTTTGTAGTTGCTTTCTGTCTGTCTGGGCTCTCTATGCACTAGAATTCTCTTTTTCCAGCATGTTTAACTGCTTTAATCAGTCGTATATGGTTAATTTTGAGTGAATATTATCACTCAGAGTAATTTGCAAAACTTAAAATTTGGTAGGATTGCGAGTAAAGGGAAGAGAATTTTGCAACCCCATGAACTGCTATCTGAGTTTGAAGCCATTTCAGAGAAGAAAAAGCTTGCTGATGGGCCATTTGGTGAAGTTTTGAGGCACACCCAGGAAGCAATTGTGTTGCCTCCATGGGTGACGCTTGCTGTTCGTCCCAGGCCTGGTGTCTGGGAGTACATCCGGGTGAATGTGGATGCTCTTGCTGTGGAGGAACTCACCCCCTCCGCATTCCTCCATGTCAAGGAGGAGTTGGTTGATGGAACGTTGAATGGGAACTTTGTGTTGGAACTGGACTTTGAGCCCTTCACTGCCTCATTCCCTCGCCCTACTCTTTCCAAGTCTATTGGGAATGGTGTGGAGTTCCTGAACAGGCACCTCTCTGCTAAGATGTTCCATGACAAGGAGAGCATGCGCCCATTGCTCGACTTCCTCAGGATGCACAACTACAAGGGCAAGACCATGATGCTGAATGACAGGATCCAAAACCTTGATACCCTCCAGAACGTTCTAAGAAAGGCCGAGGAATTCCTCACTACACTCCCTGCTGACACTCCCTACTCTGACTTGGAGCACAAATTTCAAGAGATTGGTTTGGAGAGAGGGTGGGGTGACACCGCTGAGAGAGTGCTCGACATGATCCAACTCCTCCTTGACCTTCTTGAGGCACCCGATTCTTGCACCCTCGAAAAGTTCCTGGGCCGGATTCCTATGGTTTTCAATGTTGTAATCCTATCACCCCATGGTTACTTTGCCCAGGCTAATGTTCTTGGGTACCCTGACACTGGTGGTCAGGTTGTGTACATCCTTGATCAAGTCCGTGCTTTGGAGCACGAGATGCTTCAACGTATCAAGCAGCAAGGGCTTGACATTGTCCCCAGGATCCTTATTGTAACCCGTTTGCTCCCTGATGCTGTGGGGACCACCTGTGGTCAGCGTCTTGAGAAGGTCTTTGGAACTGAGCATTCTCACATTCTTCGTGTACCCTTTAGGACCGAAAAGGGCATTGTTAGGAGATGGATTTCCAGATTTGAAGTCTGGCCATACCTTGAAACTTACACTGAGGATGTTGCAAATGAAATTGCAGGAGAGTTGCAAGCAAAGCCTGACTTGATCATTGGAAACTACAGTGATGGAAACCTTGTTGCCTCCTTGTTAGCCCACAAACTTGGAGTTACACAGTGTACAATTGCTCATGCTCTGGAAAAGACCAAGTATCCAAACTCGGACATCTACTGGAAATCATTTGAGGAGAAGTATCACTTCTCGTGTCAGTTCACTGCTGATCTTATTGCCATGAACCACACCGACTTCATCATTACTAGTACATTCCAAGAGATTGCCGGAAACAAGGACACAGTAGGACAGTATGAGAGTCACACGGCTTTCACTCTTCCTGGCCTCTACCGTGTTGTTCACGGAATTGATGTTTTTGACCCCAAATTCAACATTGTCTCACCGGGAGCTGATTTGTCTATCTACTTCCCTTACACCGAGGAGAAGAAAAGGCTCACTGCCCTCCACCCTGAAATTGAAGAGTTGCTCTTCAGTGATGTCCAAAATGAGGAGCACATGTAAGGGCCTCTTCTTGTTGTATGGGTCTCTTGTTTCATTTGAAATCTGGATGAATTTGAATGAACTTATGCTCTCTCATTCTTAGTGTGACCTGGCGGTGATGATATTGTACAACTTTGTGCTGTTGACCTTAATTTTCTGCCAATCGTGTGCTAAACAGATGTGTGCTTAAGGACCGCAACAAGCCAATCATCTTCTCAATGGCAAGGCTAGACCGGGTGAAGAACATGACCGGTCTTGTTGAGTGGTATGGTAAGAACAAGAGGTTGAGAGAGCTTGTGAACCTTGTCGTGGTTGCTGGTGATCGGAGGAAGGAATCCAAGGACATAGAAGAGAAGGAAGAGATGAAGAAAATGTATGGTCTGATTGAGCAGTACAACTTGGACGGGCAGTTCAGGTGGATCTCTGCCCAGATGAACCGGGTTAGGAATGGGGAGCTCTACAGGTACATTGCAGACACCAGGGGAGCATTTGTCCAGCCCGCTTTCTATGAAGCCTTTGGGCTCACTGTGGTTGAGTCCATGACTTGTGGGCTGCCCACCTTCGCTACTTGCCATGGTGGTCCAGCTGAGATTATAGTCCACGGAAAGTCGGGCTTCCACATTGACCCCTACCATGGTGACAAGGCGGCTGAGCTTCTGGTTGATTTCTTTGAGAAGAGCCAGGCTGATCCTACTCACTGGGAGACCATTTCCAAGGGCGGATTGAAGAGAATCGAAGAGAAGTACACATGGCAGATTTACTCTGACAGGCTCCTCACACTGGCTGGTGTTTACGGTTTCTGGAAGTATGTGTCAAATCTAGACCGTCGTGAGGCTCGCCGTTACCTCGAGATGTTCTATGCCCTTAAGTACCGCAAGCTGGCCGAGTCTGTCCCTTTGGTTGTTGAGGATTGAAGAGAGCAACCCAAAGTGTCGATTCTATGGAGAGGGGAGAGGATTCTCTTCTAGTTTGTTAAAATAACTAAGTTGAGTTCTGAGATTGTAATGAAAAGTGGAAAGTCCTTTGTTTTTTCATGCATTTTTCTCTCTTTTTTCTGAGTTTTTTTGCATTGCTTTGCGGCATTGTCTTTCATTTGATTGTGGAGGGAACATCTATGTTCCGATGTGTAATGAAATCAAGAAAGAAAGAAAAGGGTTCATTTCCTCTC

***DOD***

ATTTGATGATAATTTAATAAAAATTTAAATGCATAATCATATGAAGCTATAATAAATTACAAGGGAGGAGCTACTTGATAACCTCAAATAATAATTGTTTGTAAATGACATGCCCAACAATTAATAACATTACACTAAACCAATAATAAATTACAAACATCTTGATTTATTCACCACGAAAGAGAAAATCACAATACAAACATAAACCAAAGTTGACAACACCAACATCTGGTATGACACATTGACACCACATTCACACTCACAAAACTAAATTCTAAACCCAACTTTAAAATTACAAGAAAAGACACAAATCCAACCCAAATTTAGCTTGAACTTGATGCCATCTTTTTTCCTTTATTGATTTATTATAATAAATTTATTTACGATATATAAATTCAAGTTATTTTAAGACTGATCATAAGACGATCGATCGAGTGCCAACTTCTGAGAAAGATGGTACAAGAGAAGCTCATCCCTTCTATATAAACTTGCATTTATAGTTGTGAAATTAACTCGATCAGATGGAAGTGAACTTGTAGGAGGCATAGCCAAGAGTACCATGATCCCACGTACGATAAATGAGCTCTGCCTTTGATTTTTCACCAGCTGCACCCATGGCTACATGCAACGGTAAAAAGTGTTCTGGAATTGGATGTGCTATTTTCCACCCAGAAGGTGCTTTTGTTTGATAATTATTCACATCATCGTACCTTCCATTAATGAGAGCATCCTCAAGCCATTGATCAAACTCAGCAGCCCAAGGGGCAACCCCATCAGACCAGTGTGGGGTGTCATCAGAAGGGTGCACTGCACCCCCAGACCCAATGAACAGGACCCCTTCCTCCTTGAGAGGAGCCAAAGCCCTCCCTATGTCGAAGTGGTGGCTCGCGCTTAGGTGAGGCTGGACTGAGAGCTGGCACACCGGGATGTCAGCCTCAGGGTACATGAGGCTCAGGGGCACCCATGAGCTGTGGTCGAACCCACGACTCTCGTCTAGGCTCGCTGTCTTGAACCCTCCTGCTATCAGTAGCTCCTGCACCCTTTTGGCCAGTTTTGGTGACCCTAGAGCTGGGTACTTCATCTGGAACATACAGTCAGGAACATCGCTGAAATCGTAAATGACATCAGGATGTTCACCGGCAGATACAGAAGGCACATCAGTCTCCCAGTGAGCAGAGACAACCAGGATCGACTTGGGTTTGATGGGGAAGACATTCGTCTTCCACCCCAGCAGGAAGTTCCGTGCTATGAACGACACATCGGCCAACATGGCCGGATTCCCATGAGACACATAGAAAGTCTCCTTGAACGACACTTCTTTGCCAACACCCATCTTTGCTTCTGGTGCTGCTGGTGCAATTGCTAATGACGATCATCACTGCCTCATTCTGCTTAAATAGACTTCAGAACCATAGTTGGGGGCAAAAAAAAAAAAACATAAAAAACAACTGCCCAACACATCTCCGTCACATCCTTTAA
